# Self- regeneration of Au/CeO_2_ based catalysts with enhanced activity and ultra-stability for acetylene hydrochlorination

**DOI:** 10.1038/s41467-019-08827-5

**Published:** 2019-02-22

**Authors:** Lin Ye, Xinping Duan, Simson Wu, Tai-Sing Wu, Yuxin Zhao, Alex W. Robertson, Hung-Lung Chou, Jianwei Zheng, Tuğçe Ayvalı, Sarah Day, Chiu Tang, Yun-Liang Soo, Youzhu Yuan, Shik Chi Edman Tsang

**Affiliations:** 1 Wolfson Catalysis Centre, Department of Chemistry, University of Oxford, Oxford, OX1 3QR UK; 2 State Key Laboratory of Physical Chemistry of Solid Surfaces, National Engineering Laboratory for Green Chemical Production of Alcohols-Ethers-Esters, iChEM, College of Chemistry and Chemical Engineering, Xiamen University, Xiamen, 361005 China; 3 Department of Physics, National Tsing Hua University, Hsinchu, 30013 Taiwan; 4 Department of Materials, University of Oxford, Oxford, OX1 3PH UK; 5 Graduate Institute of Applied Science and Technology, National Taiwan University of Science and Technology, Taipei, 10617 Taiwan; 6 Diamond Light Source Ltd, Harwell Science and Innovation Campus, Didcot, OX11 0DE UK

## Abstract

Replacement of Hg with non-toxic Au based catalysts for industrial hydrochlorination of acetylene to vinyl chloride is urgently required. However Au catalysts suffer from progressive deactivation caused by auto-reduction of Au(I) and Au(III) active sites and irreversible aggregation of Au(0) inactive sites. Here we show from synchrotron X-ray absorption, STEM imaging and DFT modelling that the availability of ceria(110) surface renders Au(0)/Au(I) as active pairs. Thus, Au(0) is directly involved in the catalysis. Owing to the strong mediating properties of Ce(IV)/Ce(III) with one electron complementary redox coupling reactions, the ceria promotion to Au catalysts gives enhanced activity and stability. Total pre-reduction of Au species to inactive Au nanoparticles of Au/CeO_2_&AC when placed in a C_2_H_2_/HCl stream can also rapidly rejuvenate. This is dramatically achieved by re-dispersing the Au particles to Au(0) atoms and oxidising to Au(I) entities, whereas Au/AC does not recover from the deactivation.

## Abstract (web summary)

Despite the extensive efforts to stabilize Au catalysts for industrial hydrochlorination of acetylene to vinyl chloride the deactivation is not overcome yet. Here, the authors demonstrate that the ceria promotion to Au catalysts affords enhanced activity and stability via formation of Au(0)/Au(I) as new active pairs.

## Introduction

Solid catalysts containing highly active but dispersed single metal atoms have recently been receiving considerable attention from the catalysis community^1–3^. However, their general stability against aggregation or leaching under practical dynamic conditions is of significant concern^2^. Particularly, Au atoms have shown superior activity for many diverse catalytic reactions^4–9^, but their stability under specific reaction conditions remain to be solved. So far there is not yet any practical high throughput catalytic application for Au atoms.

Direct catalytic acetylene hydrochlorination is a crucial industrial process for coal-rich places to produce vinyl chloride monomer (VCM)^10–13^. As an important material for plastics in healthcare and medical devices, the demand for VCM increased markedly during the past decades^14,15^. Mercuric containing catalysts have been used as the main commercial catalysts for the production of VCM for more than 60 years^16^. Due to the high toxicity of mercuric chloride, mercury associated pollution problems have emerged gradually from and around the industrial sites. For most countries, the use of mercury was prohibited in VCM plants after 2017, while the already existing reactors are advised to be reduced by 50% by 2020 against the 2010 level, and to achieve mercury-free when the new process becomes technically and economically feasible^17^. As a result, given the short timescale, it is imminently required to develop a practical efficient non-mercury-based catalyst that can replace the industrial mercury-based catalysts for acetylene hydrochlorination. Dispersed Au atoms on a solid are a good candidate for the first practical alternative catalyst for large scale use, since the reaction conditions are mild for Au and also present comparable catalytic properties to Hg. As early as 1985, Hutchings suggested that supported Au catalyst is active for this reaction^11^. Subsequently, he and co-workers reported a single-site Au supported on activated carbon (Au/AC) catalyst, which showed good activity in acetylene hydrochlorination with the Au(I)/Au(III) pair proposed to be the active redox species. Based on the above Au catalyst, various promoters and supports (such as CeCl_3_, TiO_2_, ZnO, ZrO, CeO_2_, Al_2_O_3_, treated AC, 13×, etc.) have been briefly studied to further improve the catalytic performance^19–30^. However, there is on-stream deactivation of such Au catalysts, combined with the expense of significant Au loading that preclude the practical use of this otherwise promising catalyst for large volume VCM production. The main reason for deactivation of the Au catalyst is thought to be the over-reduction of active Au(III)/Au(I) species to inactive Au(0) due to thermodynamically favoured auto-reduction and/or in-excess reductants (reductive impurities). As long as Au(0) or Au particle is formed, the catalyst would suffer from non-returnable and aggregation on surface leading to deactivation^28^. Using high temperature oxidation treatment may regenerate the deactivated catalyst^30^, but the development of highly active and self-regenerated Au catalysts with economically feasible low-Au content are urgently needed to fulfil the industrial requirements.

According to previous reports, chlorinated cations with a standard reduction potential greater than that of Hg(II) 0.851 V and Pd(II) 0.951 V with respect to their metals could be active for acetylene hydrochlorinaiton^11^. On this basis, the standard reduction potential couples of Au(I)/Au(0) 1.692 V, Au(II)/Au(I) 1.800 V, Au(III)/Au(I) 1.401 V and Au(III)/Au(0) 1.498 V, which showing greater reduction potentials should catalyze the acetylene hydrochlorination despite the instability of Au(II). During the reaction, all of the above Au redox pairs may catalyse the reaction simultaneously or stepwise at various stages. However, the formation of Au(0) during over-reduction is not easy to be controlled, and its generally weak interaction with the support surface may also lead to rapid aggregation and hence deactivation of the catalyst^13^. The adaptation of chelating soft donor atoms (e.g. thiosulfate, thiocyanate, thiourea, and cyanides) and later nitrogen-containing ligands (i.e. phenanthroline ligands) have been shown to be effective to reduce over-reduction of cationic Au atoms and improve the activity and stability of Au(I)/Au(III) catalyst mixture at the acceptable industrial scale^29,30^. Despite the commercial success by this approach there is still an ongoing research in searching alternative stabilization means to reduce the progressive deactivation. CeO_2_ with facile redox properties of Ce(IV)/Ce(III) of 1.720 V may not only show good potential to disperse and enhance the re-oxidation of Au(0) to Au(I) and Au(III) but may also couple with other Au species for this catalysis with faster kinetics. It is however, Au/CeO_2_ system has been rarely studied in detail in acetylene hydrochlorination especially its effects on redox chemistry of Au species and catalyst lifetime^22^. In contrast, their redox nature and metal re-dispersion in three-way catalysts has been well-documented^31^. CeO_2_ has been found to be able to easily accept, release, and transport excess electrons in a polaronic state derived from a narrow 4f-band. It is envisaged that the Au cations may dynamically break away from the Au nanoparticle to catalyse the reaction, adjacent to the metal/oxide interface under the reaction conditions.

In this work, the activity and stability of Au have been carefully studied with CeO_2_ inclusion as compared to other metal oxides with and without porous support for acetylene hydrochorination reaction. With the incorporation of a porous support, activated carbon (AC) or zeolite 13× (13×), Au/CeO_2_ catalysts are indeed found to display the highest activity in acetylene hydrochlorination among all the catalysts studied. Prolonged on-stream laboratory testing of the catalyst has also demonstrated >3000 h (125 days) stability, implying high activity and sustainability, whereas Au/AC without the inclusion of CeO_2_ has progressively deactivated within the first 500 h. In addition, highly active catalysts with Au loading contents as low as 0.1 wt% are demonstrated, reflecting material costs that may be more acceptable to industry. Excitingly, in situ X-ray absorption (XAS) and density functional theory (DFT) calculations have revealed that the kinetically more labile redox Au(0)/Au(I) pair are catalytically more active in the presence of CeO_2_ as a mediator for the acetylene hydrochlorination reaction; with the CeO_2_ facilitating Au(0)/Au(I) dispersion, regeneration and preventing Au(0) aggregation, and thus accounting for the high stability. Total pre-reduction of Au species to inactive Au nanoparticles of Au/CeO_2_/AC when placed in C_2_H_2_/HCl stream can also rapidly rejuvenate the deactivated catalyst. It is believed that this catalyst with ceria can facilitate a catalytic mechanism using Au(0)/Au(I) redox pair to alter the activity and stability in acetylene hydrochlorination, which may offer new insights for the commercialization of Au catalysts for this and other related industrial important reactions.

## Results

### Catalytic reaction

A series of 1.0 wt% Au loaded on metal oxides was prepared by impregnation of the oxide with aqueous HAuCl_4_ in H_2_O (named as Au/metal oxide). Then a 1:1 ratio of Au/metal oxides were mixed with a high surface porous material through mechanical milling (named as Au/metal oxide and porous material). Au on activated carbon (named as Au/AC) was synthesized as the ref. ^18^ for comparison.

**Fig. 1:**
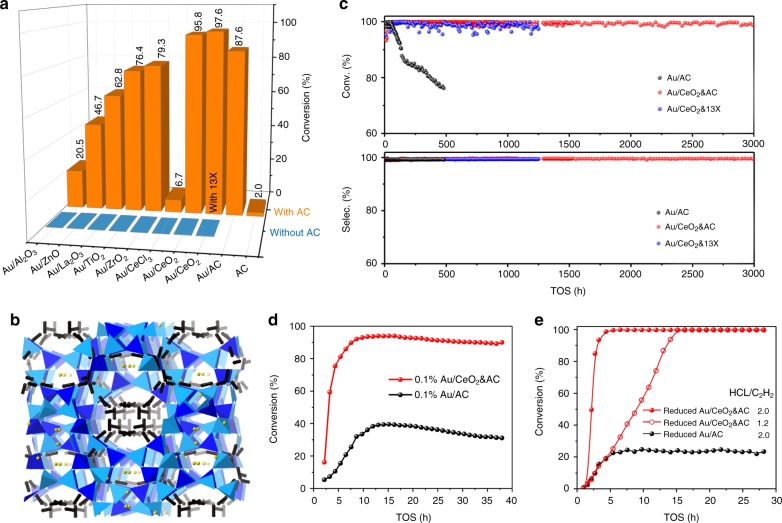
Reaction results of the acetylene hydrochlorination. **a** Various 1% Au/metal oxides and 1% Au/metal oxides and porous materials used as catalysts. [Reaction conditions: *P* = 0.1 MPa, *T* = 180 ^o^C, HCl/C_2_H_2_ = 1.2, GHSV(C_2_H_2_) = 720 h^−1^]. **b** The refined structure of the acetylene adsorbed 13X using the synchrotron X-ray powder diffraction, see Supplementary Information. **c** The stability test over Au/AC, Au/CeO_2_&AC and Au/CeO_2_&13X vs time on stream (TOS). [Reaction conditions: *P* = 0.1 MPa, *T* = 180 ^o^C, HCl/C_2_H_2_ = 1.2, GHSV(C_2_H_2_) = 60 h^−1^]. **d** The comparative activity of 0.1 wt% Au loading catalysts. [Reaction conditions: *P* = 0.1 MPa, *T* = 180 ^o^C, HCl/C_2_H_2_ = 1.2, GHSV(C_2_H_2_) = 90 h^−1^]. **e** Catalyst re-activation from pre-reduced Au/AC, Au/CeO_2_&AC and Au/CeO_2_&13×. [Reaction conditions: *P* = 0.1 MPa, *T* = 180 ^o^C, HCl/C_2_H_2_ = 1.2 or 2.0, GHSV(C_2_H_2_) = 240 h^−1^]

Figure 1a shows all of the Au/metal oxide catalysts and the pure porous materials exhibit extremely low activities (blue bar) toward acetylene hydrochlorination under this reaction condition. After mixing the Au/metal oxide with the high surface porous materials (such as: 13X and AC) (Supplementary Table 1), the activities are dramatically promoted (orange bar). Among the mixed catalysts (Fig. 1a), the Au/CeO_2_&13X and Au/CeO_2_&AC show the highest C_2_H_2_ conversion 95.8% and 97.6%, respectively. This demonstrates that CeO_2_ nanorod is a superior promotor. Under the same reaction condition, it is interesting to note that the Au/AC shows a slightly lower C_2_H_2_ conversion of 87.6% under the same loading but the selectivity of VCM is close to 99.5% for all catalysts. Both 13X and AC as support for Au are therefore active for this reaction. It is generally believed that Au surface is active for HCl dissociation, while high surface porous carbon is responsible to capture C_2_H_2_ for the materials interface to carry out efficient VCM production^10^. In order to confirm the same role of C_2_H_2_ capture by the porous support, 13X zeolite was used instead of the non-crystalline activated carbon. It is interesting to find that a high concentration of the acetylene molecules (ca. 58 molecules unit cell^−1^ equals to 6.2 kg m^−3^) can be elucidated (Supplementary Figure 1) inside the supercage using the synchrotron X-ray powder diffraction combined with Rietveld refinement (Fig. 1b and Supplementary Tables 2 and 3). This clearly indicates that the porous material indeed provides the key role in the capture of acetylene molecules in close proximity to the Au active site for HCl activation and VCM production (Fig. 1a). As stated, the stability of the catalyst is a very important aspect for its commercialization. Therefore, the long-term stability tests of the Au/AC, Au/CeO_2_&AC, and Au/CeO_2_&13X were carried under identical conditions. It is interesting to find that (Fig. 1c and Supplementary Figure 2), three catalysts show the same high C_2_H_2_ conversions of >99.9% and a VCM selectivity of >99.9% under our chosen flowing conditions within the first 70 h. However, after that, the Au/AC starts to deactivate, with the C_2_H_2_ conversion of Au/AC decreasing to 75% within the 500 h. The postmortem analysis shows that the Au particles are formed over the Au/AC sample (Supplementary Figure 3), whereas there is no sign of detectable Au particles in the case of Au/CeO_2_&13X and Au/CeO_2_&AC and they still maintain high activity without deactivation even after 1250 h (about 41 days). In comparison to Au/AC, the Au/CeO_2_&AC was allowed to continue to perform under the same conditions. It is interesting to find that Au/CeO_2_&AC maintains its activity for over 3000 h in our laboratory (about 4 months).

Considering the high cost of Au, 0.1 wt% Au loaded catalyst was also prepared and tested in this work. Figure 1d shows that 0.1 wt% Au/CeO_2_&AC displays a C_2_H_2_ conversion higher than 90%, but Au/AC at the same loading gives only 40% C_2_H_2_ conversion. The superior catalytic performance and stability of 0.1 wt% Au/CeO_2_&AC compared to Au/AC clearly shows that CeO_2_ must have displayed some crucial effects. As stated, the deactivation of the Au catalyst in acetylene hydrochlorination is ascribed to the over-reduction of Au cations to eventual Au(0) particles under an unbalanced redox reaction and/or from thermodynamically favoured auto-reduction. The additive CeO_2_ nanorod appears to maintain the high dispersion of Au(0) by presumably using its Ce(IV)/Ce(III) reduction potential of 1.720 V to re-disperse the sintered Au(0) back to Au(I) and Au(III) in an in situ manner. In order to confirm this point, a fresh 1.0 wt% Au/CeO_2_&AC and Au/AC were pre-reduced extensively to Au(0) nanoparticles under 5% H_2_/N_2_ at 350 °C for 3 h to mimic the self-regeneration of the deactivated Au(0) under the same reaction conditions (temperature programmed reduction in Supplementary Figure 4 also showed the inclusion of ceria facilitated the reduction of Au cations to Au(0) at lower temperature). Then, the reduced catalysts were transferred and tested under the reaction conditions described in supplementary methods. Interestingly, at the same ratio of HCl/C_2_H_2_, the activity of the reduced Au/CeO_2_&AC can be fully restored, while the reduced Au/AC shows only 20% C_2_H_2_ conversion recovery even after a prolonged time (Fig. 1e). Under higher ratio of 2:1 HCl/C_2_H_2_, the higher concentration of HCl as oxidant can accelerate the full recovery of the activity of Au/CeO_2_&AC within 240 min on stream (in situ X-ray absorption was performed to confirm this, see later experiments). The CeO_2_ nanorod surface appears to act as an efficient mediator that can re-disperse the sintered Au particles and re-oxidise Au(0) to Au(I) and Au(III) rapidly, which can be further demonstrated by high angle annular dark field imaging scanning transmission electron microscopy (HAADF-STEM) and X-ray absorption fine structure spectroscopy (XAFS).

### STEM characterization

**Fig. 2:**
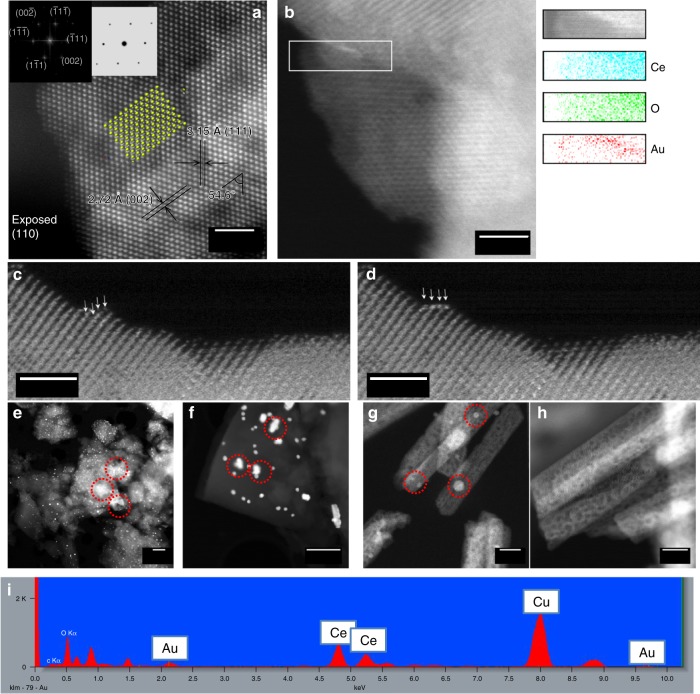
HAADF-STEM imagings and spectroscopy of the Au/CeO_2_ and Au/AC. **a** Lattice structure of an Au/CeO_2_ nanorod and annotated model structure of the CeO_2_ (110) exposed plane (yellow ball = Ce atom, light grey ball = O atom) (scale bar, 2.5 nm). Inserts are the FFT pattern and corresponding simulated FFT pattern. (also see Supplementary Figure 5) **b** Elemental EDS mapping, from the area marked within a white box, of an Au/CeO_2_ nanorod (scale bar, 2.5 nm). **c** A snapshot image of Au atoms dispersed on CeO_2_ and **d** their drift to the edge of CeO_2_ under electron beam (scale bar, 1 nm). **e**, **f** The Au/AC pre-reduced in 5% H_2_/N_2_ for 3 h and reactivated under HCl/C_2_H_2_ after 5 h (scale bar, 0.5 μm). **g**, **h** The Au/CeO_2_ pre-reduced in H_2_ for 3 h and reactivated under HCl/C_2_H_2_ after 5 h (scale bar, 10 nm). (Au particles in red cycles) **i** EDS of an Au/CeO_2_ nanorod

Figure 2a shows the HAADF-STEM images of Au/CeO_2_ nanorod (Supplementary Figure 5) with (110) facet exposed, which fits well with the crystal model of the CeO_2_ (110) plane^32^ (Fig. 2a inserted atomic model). There are no obvious Au particles buried in the CeO_2_, which would be evident as bright contrast. The lattice spacing of 3.15 Å corresponds to (111) lattice spacing and 2.72 Å to (002) spacing, with an interplanar angle of 54.6°, as shown in Fig. 2a^33^. The FFT pattern of this HAADF-STEM image is indexed (Fig. 2a inserted top-left picture), which is consistent with a simulated FFT pattern of CeO_2_ (110) plane (Fig. 2a inserted top-right picture). Thus, the major exposed facet is clearly indexed as a (110) facet. In Fig. 2b a streak of bright contrast can be seen along the edge of the CeO_2_, which EDS mapping confirms to be Au. Figures 2c, d shows four single Au atoms moving toward the CeO_2_ edge under electron beam illumination (Supplementary Movie 1 shows the relative change in atomic position between these two images). According to our DFT model, shown later, single Au atoms can dwell on this (110) surface with strong exothermicity with 4 surface oxygens. As mentioned, the activity of the extensively reduced Au/CeO_2_&AC can be fully recovered under the reaction conditions, whereas the Au/AC cannot.

Figure 2e–h gives some plausible explanation. Once the well dispersed Au atoms of Au/AC are totally pre-reduced to Au particles (Fig. 2e), most of them are unable to be re-dispersed or re-oxidised again under the reaction conditions (Fig. 2f). It means that there is strong propensity to lead to irreversible aggregation of the Au rather than its re-dispersion on the carbon surface. Therefore, the deactivation of Au/AC is taken place during the reaction as soon as over-reduction to Au(0) is reached. On the contrary, no matter how deep the reduction, the Au particles (Fig. 2g) seem to be effectively re-dispersed into Au atoms under reaction condition as no aggregated Au particles can be visually found (Fig. 2h). The presence of dispersed Au atoms on CeO_2_ nanorod was confirmed by EDS analysis (Fig. 2i). Thus, the work clearly suggests that the pre-reduced Au particles can be rapidly and extensively re-dispersed into Au single atoms on this CeO_2_ surface with the aid of HCl (Fig. 2h). According to the superior catalytic activities and stabilities of the Au/CeO_2_&AC catalysts, the re-dispersion of the Au particles and the re-oxidation of the Au(0) in the wide range of HCl/C_2_H_2_ mixture are ready to take place to give the proper balance of redox pairs (such as: Au(0)/Au(I)/Au(III)) at steady state instead of the uncontrolled aggregation of Au(0) in the case on the AC surface.

### XAFS characterization

**Fig. 3:**
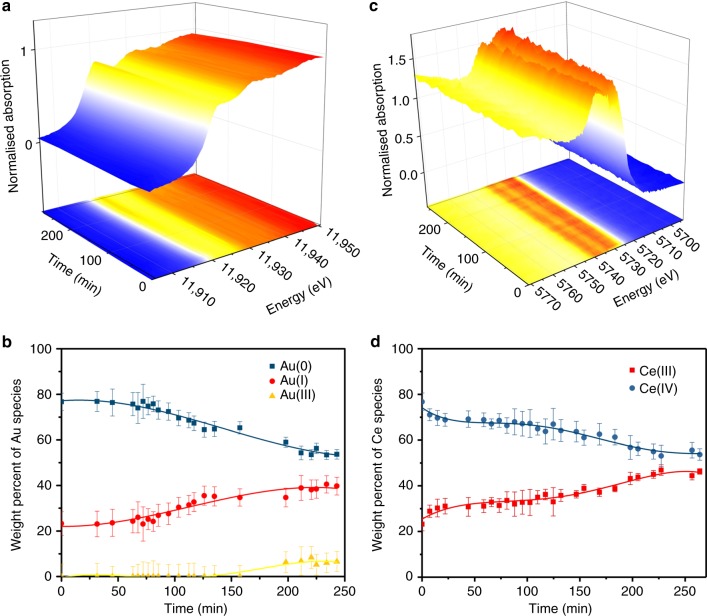
In-situ XANES of 1.0 wt% Au/CeO_2_&AC catalyst and relative fitting values. **a** The in situ XANES spectra of Au L_3_ edges as a function of reaction time and **b** the relative linear combination fitting analysis with the fitting errors. **c** The in situ XANES spectra of Ce L_3_ edges as a function of reaction time and **d** the relative multiple peak fitting analysis with the fitting errors. To minimise the conceivable influence by acetylene adsorption and the changes in metal speciation geometry in XANES spectra fitting, the higher ratio of HCl/C_2_H_2_ = 2: 1 (Fig. 1e). Au standards of 1.1 Au(III) (e.g. [AuCl_4_]^−^), 0.7 and 1.0 Au(I) (e.g. [AuCl_2_]^−^ and AuCl), and Au(0) foil, Ce standards of 1.8 Ce(IV) and 2.6 Ce(III) are used for fitting the typical normalized white-line intensity values (Supplementary Figure 6). The details of the fitting are described in supporting information. Before introducing the reactants into micro-reactor, the fresh Au/CeO_2_&AC catalyst was heated to 180 °C in N_2_ gas for 30 min. The colour indicates the difference in the white-line intensity: from 0 to 0.5 to >1 (blue to yellow to red) for Au, 0 to 0.8 to >1.5 (blue to yellow to red) for Ce

To investigate the nature of the Au and Ce oxidation state in 1.0 wt% Au/CeO_2_&AC during the reaction, in situ and ex situ synchrotron X-ray absorption experiments were performed. The white-line intensity of X-ray absorption near edge spectroscopy (XANES) has been reported as a reliable method to identify Au and Ce oxidation states^34,35^, while no photoreduction occurs significantly using the synchrotron radiation^18^. By comparing with the appropriate standards (Supplementary Figure 6), the components of the different metal oxidation states that constitute to the white-line intensity can be inferred within experimental error^36^. In ex situ XANES the white-line intensity of Au (Supplementary Figure 7) in fresh Au/CeO_2_&AC catalyst is obtained at 0.98, with linear combination fitting indicating no more than 5% contribution from Au(0), 74.9% from Au(III), 25.1% from Au(I) within errors. The high Au(I) and Au(III) signals correlate to the decomposition of HAuCl_4_^37^. The multiple peak fitting^38,39^ of the white-line intensity of Ce edge showing that CeO_2_ sample under the conditions contains 73.6% of Ce(IV) and 26.4% Ce(III). To detect the nature of Au and Ce under the reaction conditions, we built a micro-reactor to perform in situ XANES analysis with HCl:C_2_H_2_ = 2:1 at 180 °C. The white-line intensity of Au L_3_ edges and Ce L_3_ edges as a function of reaction time are shown in Fig. 3a, c, meanwhile the correlated changes of the Au and Ce components have been drawn in Fig. 3b, d. Before the addition of reactants, the fresh Au/CeO_2_&AC catalyst was heated to 180 °C in N_2_ gas. Then, the white-line intensity of Au decreased to 0.72 (0.98 at room temperature), which are contributed from 76.7% Au(0), 23.3% Au(I) and no more than 5% Au(III) within errors (Fig. 3b). Thus, this corresponds to a significant increase in the Au(0) due to auto-reduction of high oxidation states of Au species under high temperature. Interestingly, we record an oxidation process in the CeO_2_ material as its Ce(III) component has correspondingly been oxidised to Ce(IV) as compared to the fresh sample during this heating period. After 30 min isotherm at 180 °C, the reactants were introduced into the micro-reactor. During the reaction beyond the short induction period, the component of Au(I) increases almost linearly at the expense of Au(0) and there is only a much smaller increase in Au(III) concentration unless the Au(I) reaches over 35% (Fig. 3b) before they are levelled at about 240 min (while the selectivity for vinyl chloride is progressively increased, as shown in Fig. 1e). This suggests that the initial direct route for the formation of Au(I) from Au(0) is kinetically more facile than that of the direct route of Au(III) from Au(0) or indirect route of Au(III) formation via Au(I) despite the fact that all the routes are thermodynamically favourable under the reaction conditions (Supplementary Table 4). Hutchings and co-workers reported the Au(I)/Au(III) pair as the active centre for VCM production over their Au/AC. If this route exists, it could not be more dominant over Au(0)/Au(I) mediated by ceria in our case otherwise Au(III) concentration should have followed the Au(I) concentration more closely in the Fig. 3b. In addition, we also note that a corresponding dynamic reduction process on CeO_2_ material is taken place by seeing the progressive increase in the component of Ce(III) at the expense of Ce(IV) (Fig. 3d) while the simultaneous oxidation process of Au(0) to Au(I) occurs (Fig. 3b) with all curves levelling at about 240 min. Notice the fitted initial slope of Ce(III) formation from Ce(IV) of 0.078 ± 0.006 (reduction, Fig. 3d) matches well with that of the fitted initial slope of Au(I) formation from Au(0) of 0.088 ± 0.012 (oxidation, Fig. 3b) within 10% of experimental error, as shown in Supplementary Figure 8. There is a slightly higher rate for the disappearance of Au(0) (slope = −0.107 ± 0.009) than the rate of Au(I) formation especially at the increasing Au(I) concentration at later time. This is likely attributed by the contribution from the conversion of Au(0) to Au(III) (slope of formation = +0.021 ± 0.007) via Au(I) through the consecutive linear regressions of these species but this rate is relatively low. Thus, the faster Au(0)/Au(I) couple mediated by CeO_2_ material than that of the Au(I)/Au(III) pair endorses to our previous observation that this route gives higher activity in Au/CeO_2_&AC than Au/AC under identical Au loading (refer to Fig. 1a, d). We attribute this observation to the one electron fast complementary redox coupling reaction between Au(0)/Au(I) and Ce(IV)/Ce(III) in this composite catalyst whereas the 2-electron redox change of Au(I)/Au(III) would require two one-electron transfer steps from nearby Ce(IV)/Ce(III) sites with anticipated higher activation barriers. Notably, EXAFS derived Au coordination number with prolonged data acquisition under ex situ conditions are also consistent with the trend of XANES (Supplementary Figure 9 and Table 5).

As a result, it is clear that the inclusion of ceria not only can stabilize Au species from aggregation but can also facilitate a new redox couple pair namely, Au(0)/Au(I) for catalysis reaction.

### Determination of rate, activation barrier, and kinetics

**Fig. 4:**
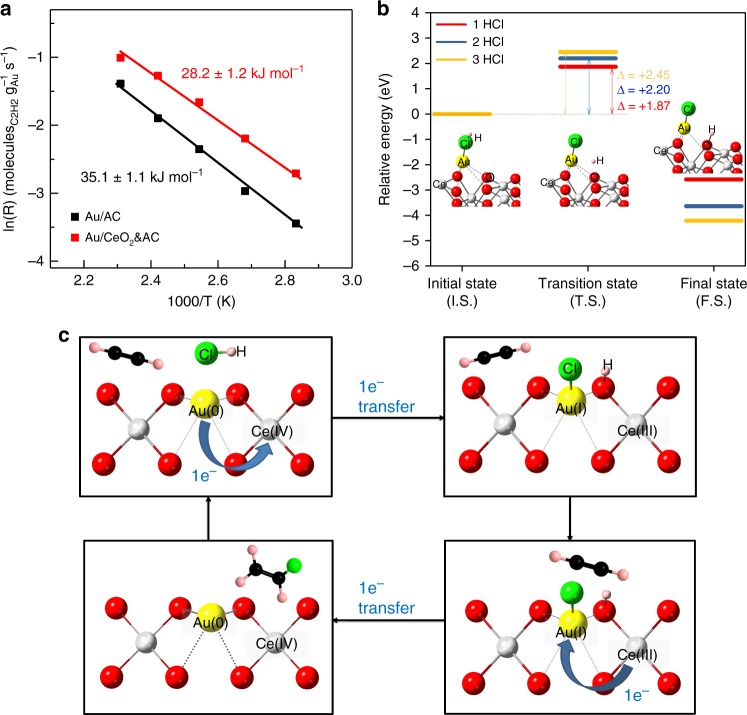
Experimental and theoretical catalytic activity. **a** Arrhenius plots for 1.0 wt% Au/AC and 1.0 wt% Au/CeO_2_&AC (the number after ‘ ± ’ represents the error). **b** The potential energy diagrams for 1HCl, 2HCl, and 3HCl molecules on Au/CeO_2_ (110) surface with the optimized geometry of the initial state, transition state, and final state, respectively (Supplementary Figure 10). Oxygen, cerium, gold, chloride, and hydrogen are depicted in red, white, yellow, green, and grey, respectively. **c** The Au(0)/Au(I) redox switching mediated by Ce(IV)/Ce(III) over Au/CeO_2_&AC in acetylene hydrochlorination to vinyl chloride

As shown in Fig. 4a, the derived activation energy from experiments is 28.2 kJ mol^−1^ over 1.0 wt% Au/CeO_2_&AC and 35.1 kJ mol^−1^ over 1.0 wt% Au/AC. Thus, the rate of acetylene hydrochlorination is indeed facilitated on Au/CeO_2_&AC than on Au/AC under the same reaction conditions. According to previous studies, the dissociation of hydrogen chloride on the catalyst surface is slow and hence is rate-limiting in acetylene hydrochlorination^40^. To investigate the elementary steps for the dissociation of hydrogen chloride over Au/CeO_2_, density functional theory (DFT) calculations using VASP (Vienna ab initio simulation package) were performed to estimate the potential dissociation energies for 1HCl, 2HCl, and 3HCl over CeO_2_ (110) plane. It is noted that only one Au atom was used in this model as shown in Supplementary Figure 10. So, we calculated the subsequent dissociation of three HCl molecules on the same stabilized Au atom by CeO_2_. First, a CeO_2_ model (10.822 × 7.652 × 21.652 Å) with exposed (110) plane was constructed. When a free Au atom was placed over the CeO_2_ (110) plane, it converged to give a strong adsorption energy of −2.1 eV with the Au atom located at the top-middle of four oxygens of the CeO_2_ (110) plane, as shown in Fig. 4c. The subsequent energy profiles for HCl dissociations and the geometries of the complexes are shown in Fig. 4b. As observed in the initial state (I.S.), the hydrogen chloride is adsorbed on Au through its Cl atom. At the transition state (T.S.), the H atom of HCl leaves the Cl (distance) and moves to the O atom of CeO_2_, as described in Fig. 4b. The highest energy barrier between T.S. and I.S. is 1.87 eV, which is the first activation barrier of the dissociation of the HCl. Consequently, the dissociation energies for the second (2.20 eV) and third (2.45 eV) HCl dissociations were found to increase, presumably due to the steric effects of placing further HCl molecules to the same Au in this configuration. Despite the differences in thermodynamic values between Au redox pairs, the calculations clearly show that the lowest kinetic barrier for the 1st HCl dissociation on Au(0) to Au(I) mediated by neighbour Ce(IV) is indeed the more facile step than that of the stepwise dissociations to produce higher oxidation states Au species, which are consistent with our experimental observations (Fig. 3b) and the rate analysis. Thus, the intrinsic higher rate for one HCl molecule dissociation to give Au(0) to Au(I) oxidation mediated by one electron complementary Ce(IV)/Ce(III) reduction at the (110) plane is clearly demonstrated to be more favourable compared to the more energetic three HCl dissociations in the conversion of Au(0) to Au(III) of non-complementary electron reduction steps. We anticipate a similarly higher activation barrier for the subsequent oxidation of Au(I) to Au(III) as in the case of Au/AC although different catalyst (Au/CeO_2_&AC) was demonstrated for calculations. The catalytic cycle between Au(0)/Au(I) mediated by Ce(IV)/Ce(III) is thus kinetically more favoured than Au(I)/Au(III) rather than their predicted trends from Au(I)/Au(II), Au(I)/Au(III) and Au(0)/Au(III) according to standard thermodynamic electrode potentials. The final state (F.S.) is the complete bond-breaking of HCl; the Cl interacts with Au and the H bonds to O of CeO_2_ and reaching the stable configuration with the lowest potential energy to this facilitated the hydrochlorination process. It should be noted that according to Fig. 4c that Au appears to remain as Au(0) in the transition state of Au(0)-Cl^−^ and Ce(IV)-OH^+^ (TS) before forming the stable products (FS) of Au(I)-Cl and Ce(III)-OH, this is not a stable state but as a concerted steps of dissociation of HCl and probably a simultaneous electron transfer to surface Ce^4+^ to Ce^3+^.

## Discussion

Combined XANES, EXAFS, and catalytic results, the proposed mechanism for the reaction of acetylene hydrochlorination is thus summarised in Fig. 4c. The thermodynamic force for fast Au(0)/Au(I) as active redox pair for hydrochlorination of acetylene is obtained from the coupling with the high redox potential of Ce(IV)/Ce(III) and the facile kinetics for the stabilization and dispersion of Au species by favourable CeO_2_ surface site. The Ce(IV) facilitates the rapid dispersion and facile oxidation of Au(0) before its aggregation to nano-clusters/nanoparticles to Au(I) on the (110) CeO_2_ surface under the reaction conditions, which avoids the classical deactivation of the Au cation to aggregated Au(0) particles on the carbon surface. As the scheme shows the redox couple is Au(0)Ce(IV)/Au(I)Ce(III) over Au/CeO_2_&AC system and the hydrochlorination initiates from the dissociation of HCl at the interface of Au/CeO_2_. As a result, the H^+^ bonds to the O of Ce(IV) and Cl^−^ interacts with the Au(0) on the CeO_2_ (110) plane. Consequently, the Au(0) is rapidly oxidized to Au(I) by the Ce(IV) being in close proximity, which will be simultaneously reduced to Ce(III), thus concomitantly increase the ratio of Ce(III)/Ce(IV) and Au(I)/Au(0) as according to the in situ XANES data. When acetylene from the reaction mixture is in contact, Au(I)-Cl and H^+^ are reductively regenerated to Au(0) with the production of VCM, which are mediated by the rapid and facile oxidation of Ce(III) in the same stable site, yielding the high activity without deactivation. Thus, we show for the first time that the Au(0) do not necessarily aggregate into an undesirable deactivated state, as seen in the Au/AC system^28^, but can be directly involved in a fast redox catalysis for VCM production due to the facile mediator roles of CeO_2_ under HCl/acetylene flow. This opens up new search for other surface mediators to utilize Au(0) directly in catalysis and eliminate its strong propensity of aggregation in complementary to the existing hunt for stabilizing higher Au species against auto-reduction.

In conclusion, we show the strong surface stabilization and fast electron redox mediating effect of CeO_2_ in maintaining the high dispersion of Au species and buffering the redox properties so no inert Au particles can be made under a wide range of HCl/acetylene ratios and reaction conditions. A faster Au(0)/Au(I) pair mediated by Ce(IV)/Ce(III) can be facilitated instead of the non-mediated Au(I)/Au(III) pair in the hydrochlorination of acetylene to VCM. The essential components for Au and CeO_2_ and porous support with lower Au content can sustain higher intrinsic hydrochlorination activity than that of Au/AC without noticeable deactivation for at least over 3000 h that may provide important clues for the commercialization of this process.

## Method

### Preparation of Au/metal oxide and porous material

A series of 1.0 wt% Au loaded on metal oxides was prepared by impregnation of the oxides with aqueous HAuCl_4_ in H_2_O (named as Au/metal oxide). Then the 1:1 ratio of Au/metal oxides were mixed with a high surface porous material through mechanically milling (named as Au/metal oxide and porous material). Similarly, ultra-low Au loading of 0.1 wt% Au/CeO_2_ was prepared based on the above procedures.

Typically, ceria oxide was prepared by hydrothermal synthesis as established methods^41^ as follows: 20 mL of 3.47 g Ce(NO_3_)_3_·6H_2_O aqueous solution and 140 mL of 9 mol L^−1^ NaOH solution were stirred for 30 min at room temperature in a 200 mL Teflon-lined stainless-steel autoclave. The sealed autoclave was transferred to a temperature controlled oven at 100 °C and held there for 24 h. After cooling, the precipitate was filtered, washed with hot deionized water and dried at 110 °C for 12 h. The obtained yellow powder was then calcined at 550 °C in air for 6 h. The activated carbon used in this study was the coconut activated carbon with a BET surface area of 1128 m^2^ g^−1^, total pore volume and average pore diameter of 0.45 cm^3^ g^−1^ and 2.68 nm, respectively. The carbon used as the milled materials was identical to the activated carbon used for other preparations.

1% Au/CeO_2_ shown in Fig. 1a was prepared by deposition-precipitation method, using urea as precipitant^42^. Typically, an aqueous solution of HAuCl_4_·4H_2_O (2.4 mL, 10 g L^−1^) was mixed with 2.0 g of CeO_2_. Then a corresponding amount of urea in a molar ratio of gold to urea of 1/125 was added as the precipitating agent. The mixed solution was stirred at 90 °C for 8 h and then aged for 12 h at room temperature. Afterward, the precipitate was separated by centrifugal, washed with deionized water and dried at 110 °C and the obtained mixture was denoted as Au/CeO_2_ (1.02 wt% Au was confirmed by inductive coupled plasma atomic emission spectrometry (ICP-AES)). Then a 1:1 ratio of Au/metal oxide was mixed with a high surface porous material through mechanical milling (named as Au/metal oxide and porous material).

### Preparation of Au/AC

AC support was initially refluxed under stirring with HNO_3_ (15 wt%) for 5 h at 80 °C to remove Na, Fe, and Al contaminants, which were the main impurities in the catalyst preparation and catalytic acetylene hydrochlorination reaction. The purified carbon sample were washed by deionized water, and dried at 120 °C overnight^43^.

Gold supported on AC samples (same type as above) were prepared via the impregnation method as previously reported^44^. For example, the 2 g Au/AC (1.0 wt% Au content) catalyst was manufactured using an incipient wetness impregnation method with the addition of aqua regia of HAuCl_4_ solution (2.4 mL, 10 g L^−1^). Then the sample was dried at 110 °C overnight and preserved as catalyst Au/AC for evaluations.

### Reactor setup

Catalytic performance of acetylene hydrochlorination was tested in a fixed bed micro-reactor (i.d. of 8 mm, BetterWorks Intelligent Technology Company) at slightly above ambient pressure. The reaction temperature was controlled by a LWT-800 temperature controller. C_2_H_2_ and gaseous HCl were dried and dehydrated through silica-gel drier. In detail, the temperature of the reactor was firstly ramped to 150 °C and then maintained there for 30 min with N_2_ flow prior to the catalytic test to remove moisture and air from the catalyst system. Afterward, the reactor temperature was adjusted to 180 °C under the flow of dried HCl (with N_2_ balance) to pre-activate the gold-based catalysts for 30 min. When the temperature of reaction system reached 180 °C, the calibrated HCl and C_2_H_2_ gases were allowed to purge into the reactor by mass flow controllers with a given GHSV(C_2_H_2_) ranging from 60 to 900 h^−1^. The pressure of the reactants was set in the range of 1.1–1.2 bar. The product gas mixture was passed to a vessel filled with NaOH solution to remove the redundant HCl and the remaining gas mixture was then analyzed by an online gas chromatography (GC-2060) equipped with a packed column (GDX-502) and a flame ionization detector. The HCl/C_2_H_2_ molar ratio of 1.2 was used^45^.

### Conversion and selectivity calculation (equation)

The concentration reactants and products were determined by our on-line system. Specifically, the analysis conditions: 4 m × Φ3 mm chromatographic column packing with GDX-502; column temperature and vaporizer temperature were controlled at 100 °C and 200 °C, respectively. The catalytic performance was evaluated in terms of acetylene conversion (*X*_*A*_) and selectivity towards the VCM (*S*_VCM_) product. The catalytic results were defined and expressed as the following equations, respectively.

1 $$X_A = \frac{{{\mathrm{\varphi }}_{{\mathrm{A}}0} - {\mathrm{\varphi }}_{\mathrm{A}}}}{{{\mathrm{\varphi }}_{{\mathrm{A}}0}}} \times 100{\mathrm{\% }},$$

2 $$S_{{\rm VCM}} = \frac{{{\mathrm{\varphi }}_{{\mathrm{VC}}}}}{{(1 - {\mathrm{\varphi }}_{\mathrm{A}})}} \times 100{\mathrm{\% }},$$

where *φ*_A0_ is designated as the volume percentage of acetylene in the mixture of reactant gases, while the *φ*_A_ and *φ*_VC_ are designated as the unreacted acetylene and produced vinyl chloride in the flow of reacted gases, respectively^46^.

Kinetic data were collected over our microreactor catalyst system with analyzing the activation energies for both the catalysts Au/C and Au/CeO_2_&AC at low C_2_H_2_ conversions of below 20% in a high GHSV so the data were far from those values at thermodynamic equilibrium and there was no substrate depletion over these temperatures.

### Laboratory characterization

Powder X-ray diffraction (XRD) diffraction patterns were examined using a Rigaku Ultima IV X-ray diffractometer fixed with Cu-K_α_ radiation (40 kV and 30 mA) scanned ranging from 10° to 90°. The diffraction characteristics were confirmed with reference features in the JCPDS cards.

The specific surface area of various catalyst systems were measured using the Brunauer–Emmett–Teller (BET) method. The total pore volume was calculated dependent on the adsorbed N_2_ volume. The average pore diameter was determined by desorption isotherm branch derived from the Barrett–Joyner–Halenda (BJH) method.

Scanning transmission electron microscopy (TEM), high-resolution TEM (HRTEM) visualization were carried out on a Philips Analytical FEI Tecnai 30 electron microscope conducted at an accelerated voltage of 300 kV. In particular, the catalyst powders were lightly ground and then dispersed in ethanol coupled with ultrasonic process at room temperature. Subsequently, the obtained solution was dropped onto the copper grids hosted by holey carbon films. The morphologies, metal nano-clusters, and microstructures of series of catalysts were imaged through a JEOL ARM200CF microscope examined at 200 kV. Analogously, TEM specimens were proceed by pipetting 5 µL catalyst solution in ethanol onto carbon-coated copper mesh grids (400 meshes).

Hydrogen-temperature-programmed reduction (H_2_-TPR) measurements were performed by a Micromeritics ASAP AutoChem II 2920 apparatus with the analysis of a thermal conductivity detector (TCD) mode. Specifically, 50 mg of catalyst was firstly pretreated in a quartz U-tube reactor at 120 °C to remove moistures and air for 2 h under the flow of He (30 mL min^−1^). Then the temperature of sample was cooled down to 50 °C protected by flowing He, afterward, the system temperature was heated from 50 to 600 °C (10 °C min^−1^) with a 5.0 vol% H_2_/Ar stream at a rate of 30 mL min^−1^.

The loadings of Au were measured by inductively coupled plasma atomic emission spectrometry (ICP-AES) with an NCS Plasma1000. The Au/CeO_2_ was added into aqua regia and boiled for 20 min. The solution was filtered into a 25 mL volumetric flask after cooling down and similarly diluted with 5% HCl solution. The catalysts with carbon supports were calcined in a muffle furnace at 550 °C for 4 h at a heating rate of 4 °C min^−1^ to burned away the carrier. The remained Au was dissolved in aqua regia and then diluted with 5% HCl into a 25 mL volumetric flask.

### Synchrotron X-ray powder diffraction (XRD)

High resolution Synchrotron PXRD data were collected on Beamline I11, Diamond Light Source, UK. Detailed description of the beamline can be found elsewhere^47^. The energy of the incident X-ray beam was set at 15 keV. The wavelength and the 2θ-zero point correction were refined using a diffraction pattern obtained from a high quality silicon powder (SRM640c). For room temperature, the fine zeolite powder was loaded in a 0.7 mm borosilicate glass capillary.

Rietveld refinement was performed using TOPAS-Academic 5. 13X zeolite starting model is obtained from reference^48^. For butene adsorbed 13X sample, SXRD peaks appear at below 2θ = 50^o^, thus data at 2.5–50^o^ was used for Rietveld refinement. In total, there are 765 *hkl* reflections measured within this 2θ range, of which at least 90-independent ones were observed. From a mathematical perspective, the number of variables should not exceed the number of observables. In the Rietveld refinement performed in this work, the number of varied structural parameters has not exceeded 60 (<90). Thus, the resulting crystallographic models are reliable. The background was described by a shifted Chebyschev function. A Thompson-Cox-Hastings pseudo-Voigt peak function was used to describe the shape of diffraction peaks. The scale factor and lattice parameters were allowed to vary at all times. Refined structural parameters include the fractional coordinates (*x*, *y*, *z*), isotropic displacement factors (*B*_eq_), site occupancy factors (SOFs), the translation and rotation of the axes of the rigid bodies describing the guest molecules within the zeolite framework. The rigid bodies were described by Z-matrix. The quality of the refinement was assured by a small weighted-profile *R*-factor (*R*_wp_), a small goodness-of-fit (GOF) factor and acceptable *B*_eq_ within experimental errors.

The crystallographic data and refinement details of all samples are summarized in Supplementary Table 2. The atomic arrangements of these samples are presented in Supplementary Table 3.

### Synchrotron X-ray absorption fine structure (XAFS)

XAFS spectra for all the Au/CeO_2_&AC samples were recorded at the Au L_3_ and Ce L_3_ absorption edge, in fluorescence mode using a Lytle fluorescence detector, under reaction conditions, at beamline BL07A of the Taiwan light source at National Synchrotron Radiation Research Center in Taiwan. A Si (111) Double Crystal Monochromator(DCM) was used to scan the photon energy. The energy resolution for the incident X-ray photons was estimated to be 2 × 10^−4^. The Demeter software package (Athena and Artemis) was used for XAFS data analysis for both the Au and Ce data. To ascertain the reproducibility of the experimental data, at least two scan sets were collected and compared for each ex situ sample. The spectra were calibrated with foils as a reference. And the amplitude parameter was obtained from EXAFS data analysis of the Au foil, which was used as a fixed input parameter in the data fitting to allow the refinement in the coordination number of the absorption element. In this work, the first shell data analyses under the assumption of single scattering were performed with the errors estimated by R-factor.

The X-ray near edge structure (XANES) region of the XAFS spectrum is used to probe and quantify the relative ratio of different oxidation states. For Au the white line intensity could be interpreted as the primary transition from Au 2p_3/2_ to 5d orbital. Therefore the white line intensity is in a direct correlation to the oxidation state. A linear combination fitting is employed using standards of AuCl_3_, [AuCl_2_]^−^, AuCl, and Au foil (Supplementary Figure 6).

As for Ce a quantitative analysis was performed by simulating the edge jump with arctangent function and the peak features with Gaussian function^49^. The centre of the arctangent function was set as the inflection point of the main absorption edge. Peak positions at 5737.7 eV and 5730.8 eV were assigned to the final state 2p_h_4f^0^5d* and 2p_h_4f^0^5d*L_h_ with reference from a CeO_2_ standard, where p_h_ represents the electron hole resulted in the 2p_3/2_ orbital, 5d* denotes the presence of the excited electron in the 5d orbital, and L_h_ refers to the ligand hole present in the anion orbital. Peak position at 5726.3 eV was assigned to the valence state of Ce^3+^ with reference from a CeCl_3_ standard. A small peak at 5720.5 eV in the pre-edge region was also assigned to the final state 2p4f* which is forbidden due to the selection rule. The individual peak areas from Ce^3+^ and Ce^4+^ were integrated and the corresponding weight ratio is calculated. The R-factor obtained (<0.06%) is within acceptable value. (Supplementary Figure 6).

For the in situ measurements the spectrum was obtained in fluorescence mode using a fixed bed reactor with Kapton windows to allow synchrotron X-rays to pass through. The temperature was controlled with a Eurotherm controller with a thermocouple positioned in the centre of the heating block. For safety reasons, dilute gas mixtures were used. C_2_H_2_/Ar (5% balanced in N_2_, BOC) and HCl/Ar (5% balanced in N_2_) gases were introduced to the heated chamber containing the fixed bed of catalysts with flow rates controlled by mass flow controllers. The reactor was heated to 180 °C at a ramp rate of 5 °C/min and held at temperature for 30 min under a flow of N_2_. The reaction gas HCl/Ar was first introduced into the system at a flow rate 40 mL min^−1^ and XAFS spectrum was obtained until no change in white line intensity was observed. N_2_ (40 mL min^−1^) was then passed to clean the surface and held at 30 min. The reaction gas C_2_H_2_/Ar was then subsequently introduced into the system at a flow rate 40 mL min^−1^ and XAFS spectrum was obtained until no change in white line intensity was observed. N_2_ (40 mL min^−1^) was then passed to clean the surface and held at 30 min. Finally mixed gas HCl/Ar (40 ml min^−1^) and C_2_H_2_/Ar (20 mL min^−1^) was passed. The time resolution of the data acquisition for Au and Ce was 4 min 30 s and 7 min 20 s, respectively.

### DFT calculation

To investigate the elementary steps for the dissociation of the hydrogen chloride over the Au/CeO_2_, density functional theory (DFT) calculations using VASP were performed to estimate the potential dissociation energies for 1HCl, 2HCl, and 3HCl over CeO_2_ (110) plane. First, a CeO_2_ model (10.822 × 7.652 × 21.652 Å) with exposed (110) plane was constructed. The periodic slab model comprised a five-layer CeO_2_, a supercell with dimensions of 10.822 × 7.652 × 21.652 Å^3^ for the 20 Ce-atom and 40 O-atoms, separated by a vacuum space of 14 Å, exposing the {110} facet; and the adsorbed HCl molecule and the Au atom above the surface, the top three-layer were allowed to fluctuate by a given perturbation, while the Ce and O atoms at the bottom two-layer remained fixed as the boundary condition. Total energy calculations were performed using a 3 × 3 × 1 k-point mesh. During the structural optimization, HCl molecule was supposed to adsorb on the Au/CeO_2_ (110) slab. The simulations were undertaken using Au/CeO_2_(110) slab to model the E_ads_ of HCl on the Au/CeO_2_(110) slabs. The adsorption energy, E_ads_ is defined as the sum of interactions between the adsorbate HCl molecule and slab atoms, and it is given as E_ads_ = E_total_−E_Au/CeO2(110) slab_–E_ligand_, where E_total_, E_Au/CeO2(110) slab_, and E_ligand_ are total energy of the system, Au/CeO_2_(110) slab energy, and adsorbate ligand energy (one HCl molecule, two HCl molecules and three HCl molecules), respectively. The negative sign of Eads corresponds to the energy gain of the system due to ligand adsorption. In DFT calculations, we employed projector-augmented waves (PAW)^50–53^ generalized gradient approximation (GGA)^54,55^ as implemented in the Vienna ab initio simulation package (VASP)^56,57^. The spin-polarized first-principles total energy calculations are performed using an ultrasoft pseudopotential method. For the transition state (TS) determination, the nudged elastic band (NEB) method^55,58–60^ implemented in VASP, was applied. The system would gradually become relaxed to achieve a balanced state with convergent energy, when the forces on the relaxed atoms were less than 0.01 eV/Å. The relative energies of the initial state, transition state, and final state presented in this study were zero-point-energy obtained from frequency calculations at the same level of optimization. The energy convergence of the I.S. and F.S. was scanned by 16 images.

## Supplementary information

Supplementary Information

Peer Review File

Description of Additional Supplementary Files

Supplementary Movie 1

## Data Availability

The authors declare that all the published data supporting the findings of this study are available within the article and its supplementary information files.
