# Supplementary material for: Self- regeneration of Au/CeO2 based catalysts with enhanced activity and ultra-stability for acetylene hydrochlorination
Source: Nat Commun. 2019 Feb 22;10:914. doi: 10.1038/s41467-019-08827-5 (PMC6385229; doi:10.1038/s41467-019-08827-5)
Supplement: Supplementary file 1 — Supplementary Information [file 41467_2019_8827_MOESM1_ESM.docx]

**Supplementary materials for**

Self-Regeneration of Au/CeO_2_ based Catalysts with Enhanced Activity and Ultra-stability for Acetylene Hydrochlorination

Lin Ye^1†^, Xinping Duan^2†^, Simson Wu^1†^, Tai-Sing Wu^3^, Yuxin Zhao^2^, Alex W. Robertson^4^, Hung-Lung Chou^5^, Jianwei Zheng^1^,Tuğçe Ayvalı^1^, Sarah Day^6^, Chiu Tang^6^, Yun-Liang Soo^3^, Youzhu Yuan^2*^ and Shik Chi Edman Tsang^1*^

**This file includes:**

Supplementary Methods

Supplementary Figures 1-10

Supplementary Tables 1-5

Supplementary Movies 1

Supplementary References 1-23

Supplementary Methods

Preparation of Au/metal oxide & porous material

A series of 1.0 wt% Au loaded on metal oxides was prepared by impregnation of the oxide with aqueous HAuCl_4_ in H_2_O (named as Au/metal oxide). Then the 1:1 ratio of Au/metal oxides were mixed with a high surface porous material through mechanically milling (named as Au/metal oxide & porous material). Similarly, ultra-low Au loading of 0.1 wt% Au/CeO_2_ was prepared based on the above procedures.

Ceria oxide was prepared by hydrothermal synthesis as established methods [^1^](#_ENREF_1). Typically, 20 mL of 3.47 g Ce(NO_3_)_3_·6H_2_O aqueous solution and 140 mL of 9 mol·L^−1^ NaOH solution were stirred for 30 min at room temperature and then added to a 200 mL Teflon-lined stainless-steel autoclave. The sealed autoclave was transferred to a temperature controlled oven at 100 ºC and held there for 24 h. After cooling, the precipitate was filtered, washed with hot deionized water and dried at 110 ºC for 12 h. The obtained yellow powder was then calcined at 550 ºC in air for 6 h. The activated carbon used in this study was the coconut activated carbon with a BET surface area of 1128 m^2^ g^-1^, total pore volume and average pore diameter of 0.45 cm^3^ g^-1^ and 2.68 nm, respectively. The carbon used for the milled materials was identical to the activated carbon used for other preparations.

Since it was found that the direct deposition of Au on ceria prepared by impregnation above was not active. As a result, 1% Au/CeO_2_ shown in Fig. 1a was prepared by the deposition-precipitation method, using urea as precipitant [^2^](#_ENREF_2). Typically, an aqueous solution of HAuCl_4_·4H_2_O (2.4 mL, 10 g·L^−1^) was mixed with 2.0 g of CeO_2_. Then a corresponding amount of urea in a molar ratio of gold to urea of 1/125 was added as the precipitating agent. The mixed solution were stirred at 90 ºC for 8 h and then aged for 12 h at room temperature. Afterward, the precipitate was separated by centrifugal, washed with deionized water and dried at 110 ºC, the obtained mixture was denoted as Au/CeO_2_ (1.02 wt% Au was confirmed by inductive coupled plasma atomic emission spectrometry (ICP-AES)).

Prepareation of Au/AC

Au on activated carbon (named as Au/AC) was synthesized as the [^3^](#_ENREF_3) for comparison.

AC support was initially refluxed under stirring with HNO_3_ (15 wt%) for 5 h at 80 °C to remove Na, Fe, and Al contaminants, which were the main impurities in the catalyst preparation and catalytic acetylene hydrochlorination reaction. The purified carbon sample was washed by deionized water, and dried at 120 °C overnight.[^4^](#_ENREF_4)

Gold supported on AC samples were prepared via incipient wetness impregnation method as previously reported.[^5^](#_ENREF_5) For example, the 2 g Au/AC (1.0 wt% Au content) catalyst was manufactured using an incipient wetness impregnation method with the addition of aqua regia of HAuCl_4_ solution (2.4 mL, 10 g L^−1^). Then the sample was dried at 110 °C overnight and preserved as catalyst for evaluations. This method was present as the most effective preparation of carbon-based material supported gold catalysts for hydrochlorination reaction.

Reactor set-up

Catalytic performance of acetylene hydrochlorination was tested in a fixed bed micro-reactor (i.d. of 8 mm, BetterWorks Intelligent Technology Company, Xiamen, China) at slightly above the ambient pressure. The reaction temperature was controlled by a LWT-800 temperature controller. C_2_H_2_ and gaseous HCl were dried and dehydrated through silica-gel drier. In detail, the temperature of the reactor was firstly ramped to 150 °C and then maintained there for 30 min with N_2_ flow prior to the catalytic test to remove moisture and air from the catalyst system. Afterward, the reactor temperature was adjusted to 180 °C under the flow of dried HCl (with N_2_ balance) to pre-activate the gold-based catalysts for 30 min. When the temperature of reaction system reached 180 °C, the calibrated HCl and C_2_H_2_ gases were allowed to purge into the reactor by mass flow controllers with a given GHSV(C_2_H_2_) ranging from 60 to 900 h^−1^. The pressure of the reactants was set in the range of 1.1–1.2 bar. The reaction gas mixture was passed through a vessel filled with NaOH solution to remove the redundant HCl and the mixture was then analyzed online with gas chromatography (GC-2060) equipped with a packed column (GDX-502) and a flame ionization detector. The HCl/C_2_H_2_ molar ratio of 1.2 was used ^6^.

Conversion and selectivity calculation (equation)

The concentration reactants and products were determined by our on-line system. Specifically, the analysis conditions: 4 m × Φ3 mm chromatographic column packing with GDX-502; column temperature and vaporizer temperature were controlled at 100 °C and 200 °C, respectively. The catalytic performance was evaluated in terms of acetylene conversion (*X_A_*) and selectivity towards the VCM (*S_VCM_*) product. The catalytic results were defined and expressed as the following equations, respectively.

$X_{A}=\frac{\varphi_{A0} -\varphi_{A}}{\varphi_{A0}}\times100\%$ (1)

$S_{VCM}=\frac{\varphi_{\mathrm{VC}}}{(1 - \varphi_{A})}\times100\%$ (2)

where $\varphi_{A0}$is designated as the volume percentage of acetylene in the mixture of reactant gases, while the $\varphi_{A}$ and $\varphi_{\mathrm{VC}}$ are designated as the unreacted acetylene and produced vinyl chloride in the flow of reacted gases, respectively ^46^.

Kinetic data was collected over our microreactor catalyst system with analyzing the activation energies for both the catalysts Au/C and Au/CeO_2_&AC at low C_2_H_2_ conversions of below 20% in a high GHSV so the data were far from those values at thermodynamic equilibrium and there was no substrate depletion over these temperatures.

Laboratory characterization

Powder X-ray diffraction (XRD) diffraction patterns were examined using a Rigaku Ultima IV X-ray diffractometer fixed with Cu-K_α_ radiation (40 kV and 30 mA) scanned ranging from 10° to 90°. The diffraction characteristics were confirmed with reference features in the JCPDS cards.

The specific surface area of various catalyst systems were measured using the Brunauer−Emmett−Teller (BET) method. The total pore volume was calculated dependent on the adsorbed N_2_ volume. The average pore diameter was determined by desorption isotherm branch derived from the Barrett−Joyner−Halenda (BJH) method.

Scanning transmission electron microscopy (TEM), high-resolution TEM (HRTEM) visualization were carried out on a Philips Analytical FEI Tecnai 30 electron microscope conducted at an accelerated voltage of 300 kV. In particular, the catalyst powders were lightly ground and then dispersed in ethanol coupled with ultrasonic process at room temperature. Subsequently, the obtained solution was dropped onto the copper grids hosted by holey carbon films. The morphologies, metal nanoclusters, and microstructures of series of catalysts were imaged through a JEOL ARM200CF microscope examined at 200 kV. Analogously, TEM specimens were proceed by pipetting 5 µL catalyst solution in ethanol onto carbon-coated copper mesh grids (400 meshes).

Hydrogen-temperature-programmed reduction (H_2_-TPR) measurements were performed by a Micromeritics ASAP AutoChem II 2920 apparatus with the analysis of a thermal conductivity detector (TCD) mode. Specifically, 50 mg of catalyst was firstly pretreated in a quartz U-tube reactor at 120 °C to remove moistures and air for 2 h under the flow of He (30 mL min^−1^). Then the temperature of sample was cooled down to 50 °C protected by flowing He, afterward, the system temperature was heated from 50 to 600 °C (10 °C min^−1^) with a 5.0 vol% H_2_/Ar stream at a rate of 30 mL min^−1^.

The loadings of Au were measured by inductively coupled plasma atomic emission spectrometry (ICP-AES) with an NCS Plasma1000. The Au/CeO_2_ was added into aqua regia and boiled for 20 min. The solution was filtered into a 25 mL volumetric flask after cooling down and similarly diluted with 5% HCl solution. The catalysts with carbon supports were calcined in a muffle furnace at 550 ºC for 4 h at a heating rate of 4 ºC min^−1^ to burned away the carrier. The remained Au was dissolved in aqua regia and then diluted with 5% HCl into a 25 mL volumetric flask.

Synchrotron XRD

High resolution Synchrotron PXRD data were collected on Beamline I11, Diamond Light Source, UK. Detailed description of the beamline can be found elsewhere[^8^](#_ENREF_8). The energy of the incident X-ray beam was set at 15 keV. The wavelength and the 2θ-zero point correction were refined using a diffraction pattern obtained from a high quality silicon powder (SRM640c). For room temperature, the fine zeolite powder was loaded in a 0.7 mm borosilicate glass capillary.

Rietveld refinement was performed using TOPAS-Academic 5. 13X zeolite starting model is obtained from reference[^9^](#_ENREF_9). For butene adsorbed 13X sample, SXRD peaks appear at below 2θ = 50^o^, thus data at 2.5–50^o^ was used for Rietveld refinement. In total, there are 765 *hkl* reflections measured within this 2θ range, of which at least 90 independent ones were observed. From a mathematical perspective, the number of variables should not exceed the number of observables. In the Rietveld refinement performed in this work, the number of varied structural parameters has not exceeded 60 (< 90). Thus, the resulting crystallographic models are reliable. The background was described by a shifted Chebyschev function. A Thompson-Cox-Hastings pseudo-Voigt peak function was used to describe the shape of diffraction peaks. The scale factor and lattice parameters were allowed to vary at all times. Refined structural parameters include the fractional coordinates (*x*, *y*, *z*), isotropic displacement factors (*B*_eq_), site occupancy factors (SOFs), the translation and rotation of the axes of the rigid bodies describing the guest molecules within the zeolite framework. The rigid bodies were described by Z-matrix. The quality of the refinement was assured by a small weighted-profile *R*-factor (*R*_wp_), a small goodness-of-fit (GOF) factor and acceptable *B*_eq_ within experimental errors.

The crystallographic data and refinement details of all samples are summarized in Table S2. The atomic arrangements of these samples are presented in Table S3.

Synchrotron X-ray absorption fine structure (XAFS)

XAFS spectra for all the Au/CeO_2_&AC samples were recorded at the Au L_3_ and Ce L_3_ absorption edge, in fluorescence mode using a Lytle fluorescence detector, under reaction conditions, at beamline BL07A of the Taiwan light source at National Synchrotron Radiation Research Center in Taiwan. A Si (111) Double Crystal Monochromator( DCM) was used to scan the photon energy. The energy resolution for the incident X-ray photons was estimated to be 2×10^-4^. The Demeter software package (Athena and Artemis) was used for XAFS data analysis for both the Au and Ce data. To ascertain the reproducibility of the experimental data, at least two scan sets were collected and compared for each ex-situ sample. The spectra were calibrated with foils as a reference. And the amplitude parameter was obtained from EXAFS data analysis of the Au foil, which was used as a fixed input parameter in the data fitting to allow the refinement in the coordination number of the absorption element. In this work, the first shell data analyses under the assumption of single scattering were performed with the errors estimated by R-factor.

The X-ray near edge structure (XANES) region of the XAFS spectrum is used to probe and quantify the relative ratio of different oxidation states. For Au the white line intensity could be interpreted as the primary transition from Au 2p_3/2_ to 5d orbital. Therefore the white line intensity is in a direct correlation to the oxidation state. A linear combination fitting is employed using standards of AuCl_3_, [AuCl_2_]^-^, AuCl and Au foil (Fig. S6).

As for Ce a quantitative analysis was performed by simulating the edge jump with arctangent function and the peak features with Gaussian functions[^10-12^](#_ENREF_10). The center of the arctangent function was set as the inflection point of the main absorption edge. Peak positions at 5737.7 eV and 5730.8 eV were assigned to the final state 2p_h_4f^0^5d* and 2p_h_4f^0^5d*L_h_ with reference from a CeO_2_ standard, where p_h_ represents the electron hole resulted in the 2p_3/2_ orbital, 5d* denotes the presence of the excited electron in the 5d orbital, and L_h_ refers to the ligand hole present in the anion orbital. Peak position at 5726.3 eV was assigned to the valence state of Ce^3+^ with reference from a CeCl_3_ standard. A small peak at 5720.5 eV in the pre-edge region was also assigned to the final state 2p4f* which is forbidden due to the selection rule. The individual peak areas from Ce^3+^ and Ce^4+^ were integrated and the corresponding weight ratio is calculated. The R-factor obtained (<0.06%) is within acceptable value. Fig. S6).

For the in-situ measurements the spectrum was obtained in fluorescence mode using a fixed bed reactor with Kapton windows to allow synchrotron X-rays to pass through. The temperature was controlled with a Eurotherm controller with a thermocouple positioned in the centre of the heating block. For safety reasons, dilute gas mixtures were used. C_2_H_2_/Ar (5% balanced in N_2_, BOC) and HCl/Ar (5% balanced in N_2_) gases were introduced to the heated chamber containing the fixed bed of catalysts with flow rates controlled by mass flow controllers. The reactor was heated to 180 °C at a ramp rate of 5 °C/min and held at temperature for 30 min under a flow of N_2_. The reaction gas HCl/Ar was first introduced into the system at a flow rate 40 mL min^-1^ and XAFS spectrum was obtained until no change in white line intensity was observed. N_2_ (40 mL min^-1^) was then passed to clean the surface and held at 30 minutes. The reaction gas C_2_H_2_/Ar was then subsequently introduced into the system at a flow rate 40 mL min^-1^ and XAFS spectrum was obtained until no change in white line intensity was observed. N_2_ (40 mL min^-1^) was then passed to clean the surface and held at 30 minutes. Finally mixed gas HCl/Ar (40 ml min^-1^) and C_2_H_2_/Ar (20 mL min^-1^) was passed. The time resolution of the data acquisition for Au and Ce was 4 minutes 30s and 7 minutes 20s respectively.

DFT calculation

To investigate the elementary steps for the dissociation of the hydrogen chloride over the Au/CeO_2_, density functional theory (DFT) calculations using VASP were performed to estimate the potential dissociation energies for 1HCl, 2HCl and 3HCl over CeO_2_ (110) plane. First, a CeO_2_ model (10.822x 7.652x 21.652 Å) with exposed (110) plane was constructed. The periodic slab model comprised a five-layer CeO_2_, a supercell with dimensions of 10.822 × 7.652 × 21.652 Å^3^ for the 20 Ce-atom and 40 O-atoms, separated by a vacuum space of 14 Å, exposing the {110} facet; and the adsorbed HCl molecule and the Au atom above the surface, the top three-layer were allowed to fluctuate by a given perturbation, while the Ce and O atoms at the bottom two-layer remained fixed as the boundary condition. Total energy calculations were performed using a 3 × 3 × 1 k-point mesh. During the structural optimization, HCl molecule was supposed to adsorb on the Au/CeO_2_ (110) slab. The simulations were undertaken using Au/CeO_2_(110) slab to model the E_ads_ of HCl on the Au/CeO_2_(110) slabs. The adsorption energy, E_ads_ is defined as the sum of interactions between the adsorbate HCl molecule and slab atoms, and it is given as E_ads_ = E_total_-E_Au/CeO2(110) slab_-E_ligand_, where E_total_, E_Au/CeO2(110) slab_, and E_ligand_ are total energy of the system, Au/CeO_2_(110) slab energy, and adsorbate ligand energy (one HCl molecule, two HCl molecules and three HCl molecules), respectively. The negative sign of E_ads_ corresponds to the energy gain of the system due to ligand adsorption. In DFT calculations, we employed projector-augmented waves (PAW)[^13-16^](#_ENREF_13) generalized gradient approximation (GGA)[^17^](#_ENREF_17)^,^[^18^](#_ENREF_18) as implemented in the Vienna ab initio simulation package (VASP)[^19^](#_ENREF_19)^,^[^20^](#_ENREF_20) The spin-polarized first-principles total energy calculations are performed using an ultrasoft pseudopotential method. For the transition state (TS) determination, the nudged elastic band (NEB) method [^18^](#_ENREF_18)^,^[^21-23^](#_ENREF_21) implemented in VASP, was applied. The system would gradually become relaxed to achieve a balanced state with convergent energy, when the forces on the relaxed atoms were less than 0.01 eV/Å. The relative energies of the initial state, transition state and final state presented in this study were zero-point-energy obtained from frequency calculations at the same level of optimization. The energy convergence of the I.S. and F.S. was scanned by 16 images.

Supplementary Figures


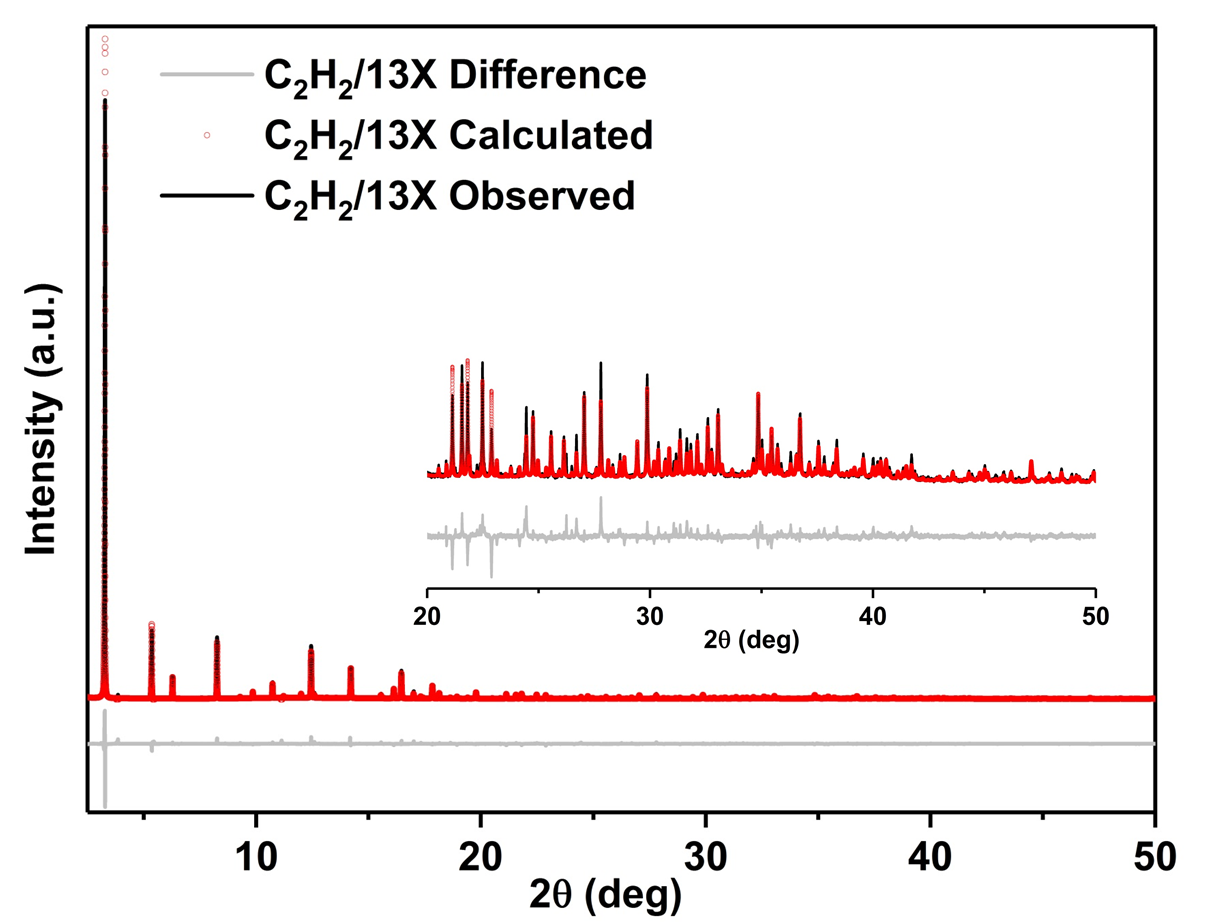


Supplementary Figure 1. SXRD of C_2_H_2_ adsorbed 13X at room temperature. Comparison of the experiment data (black line) and Rietveld refinement (red cycle) and the difference between them (grey line) for SXRD patterns of C_2_H_2_ adsorbed 13X at room temperature at a 2θ range of 2.5 - 50° and (b) 20 - 50°. During the refinement the small impurity peaks 3.77 to 3.88, 5.4 to 5.48 and 11.1 to 11.16 were excluded.


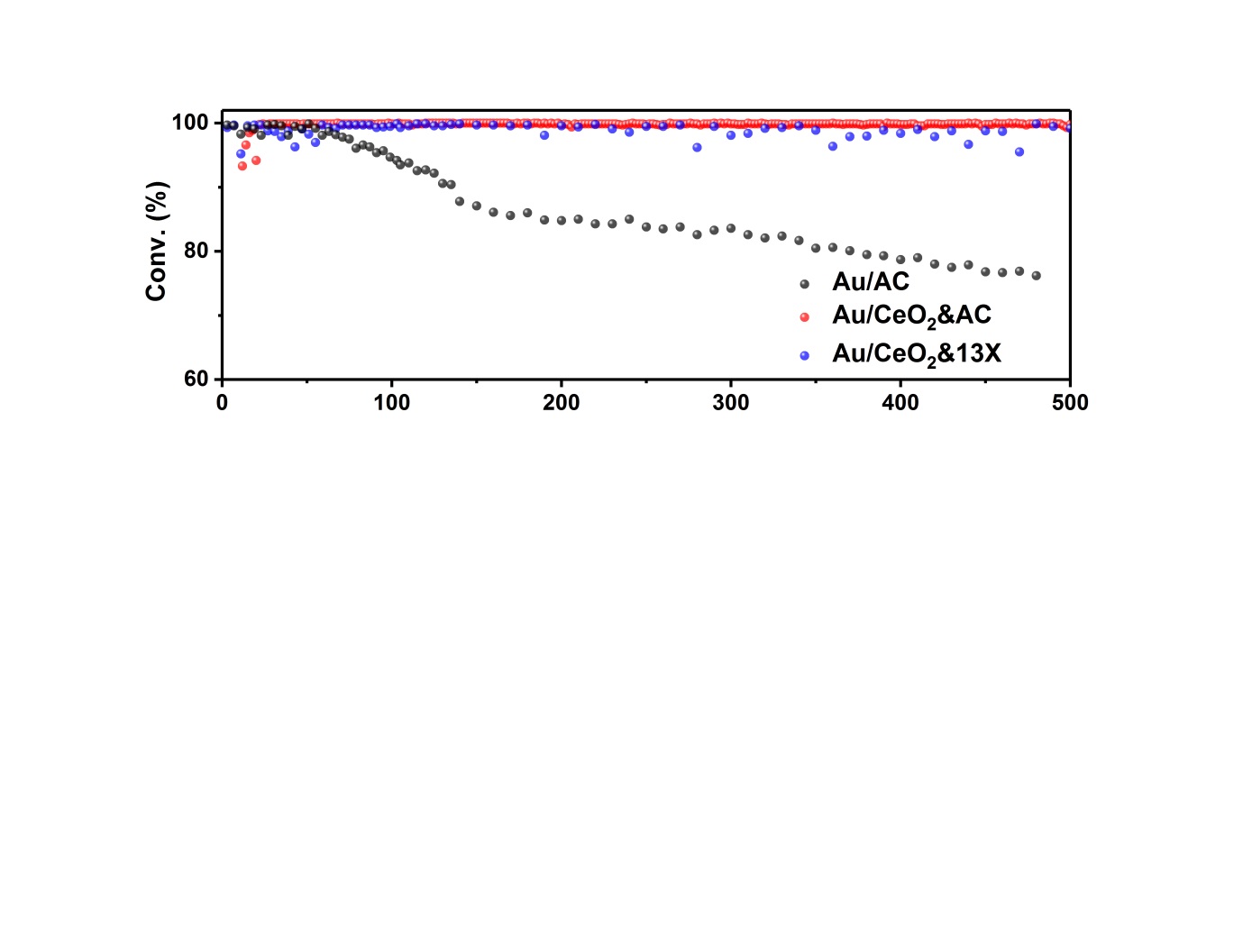


TOS (h)


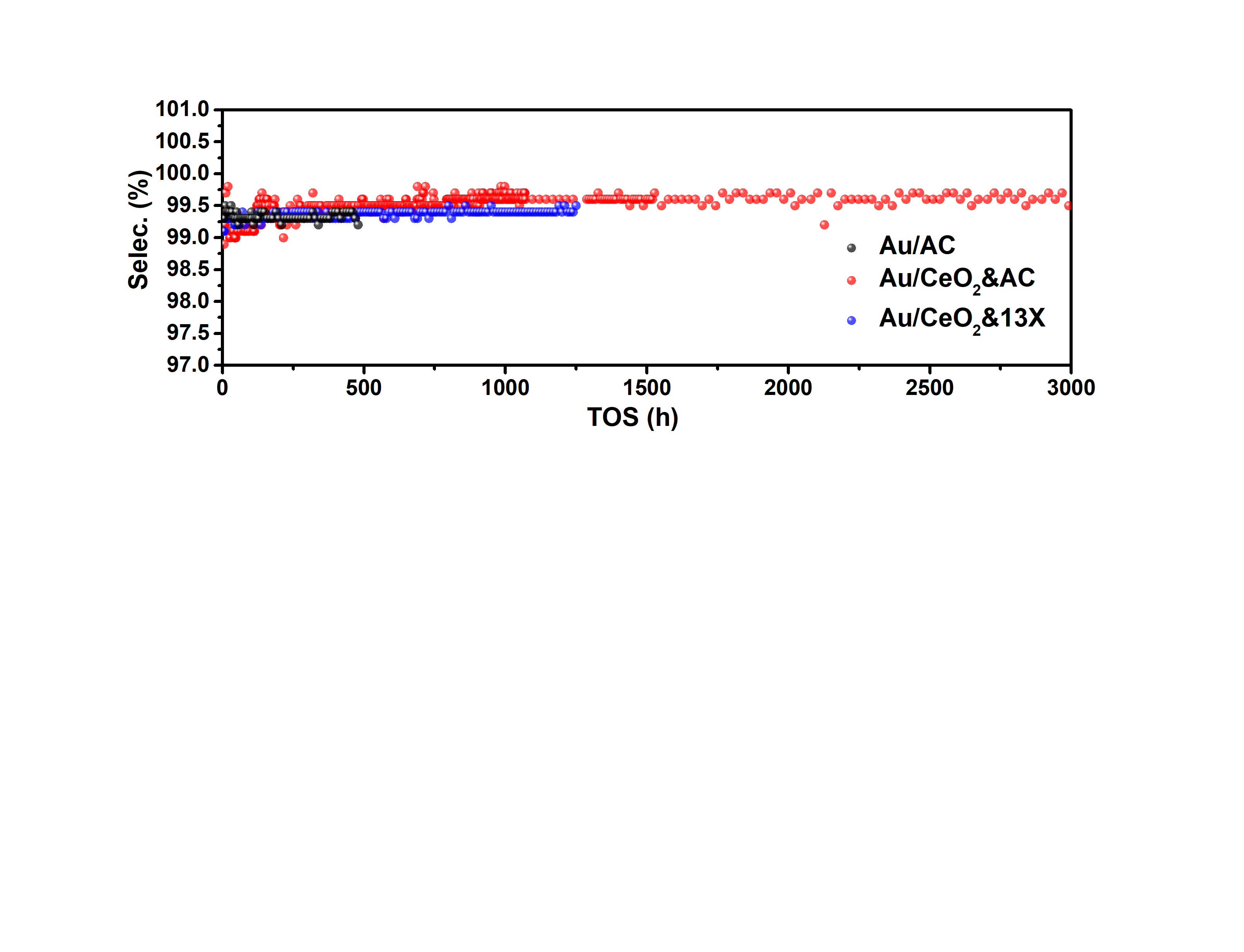


Supplementary Figure 2. The conversion change over Au/AC, Au/CeO_2_&AC and Au/CeO_2_&13X. The range from 0-150 h has been illustrated. [Reaction conditions: P = 0.1 MPa, T = 180 ^o^C, HCl/C_2_H_2_ = 1.2, GHSV(C_2_H_2_) = 60 h^-1^] (upper). The selectivity change over Au/AC, Au/CeO_2_&AC and Au/CeO_2_&13X. The range from 0-3000 hour has been illustrated. [Reaction conditions: P = 0.1 MPa, T = 180 ^o^C, HCl/C_2_H_2_ = 1.2, GHSV(C_2_H_2_) = 60 h^-1^] (bottom).


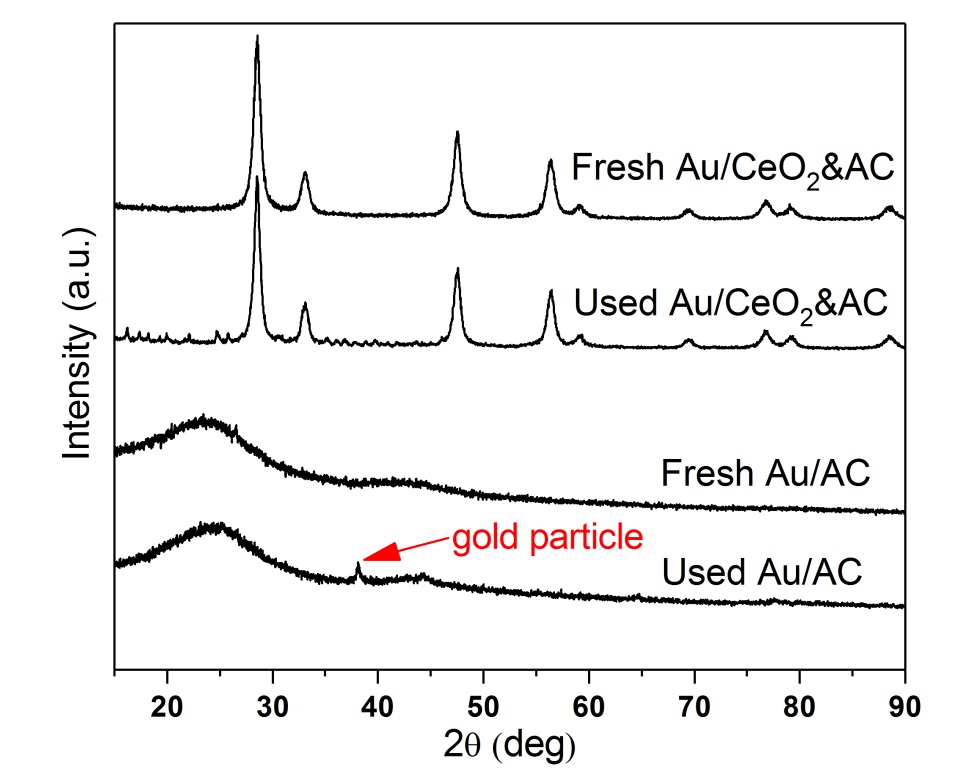


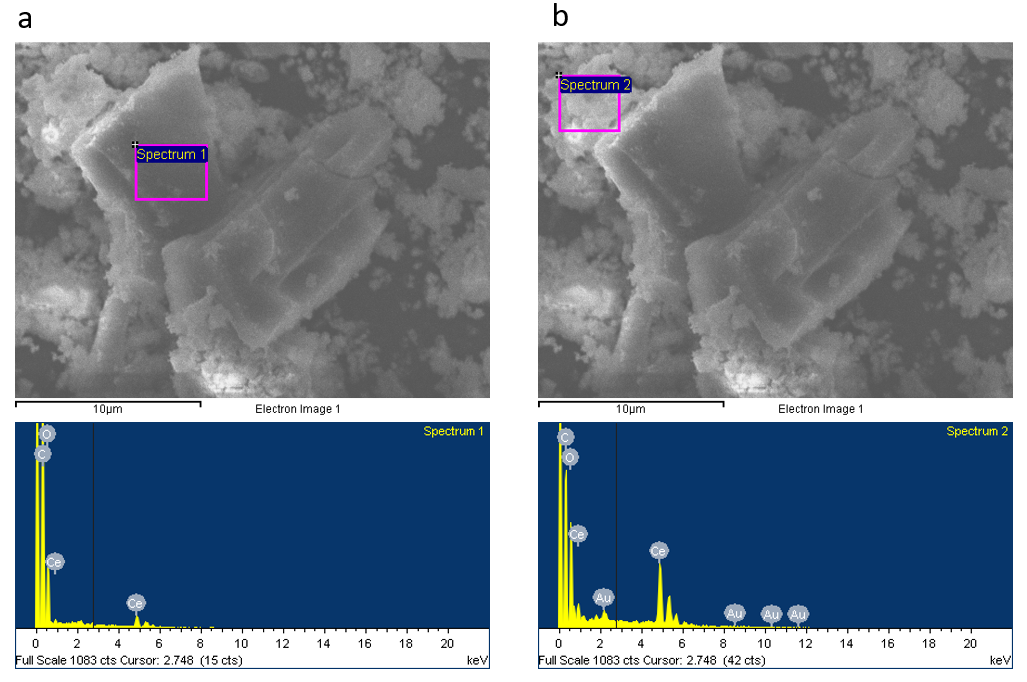


Supplementary Figure 3. Lab-source XRD patterns of the fresh and used Au/AC and Au/CeO_2_&AC. The peak at 38.1^o^ is attributed to the existence of gold particles (upper). Lab-source SEM and EDX analysis of the fresh Au/AC. Au peak is found to be fairly well-distributed on carbon (more on broken area than smooth area as shown) but no significant size of Au particle from typical TEM and SEM images can be seen. We presumed that Au atoms are equally dispersed well on AC in fresh sample but we failed to identify Au atoms on AC by HAADF-STEM (as that of single atoms on ceria in Fig. 2c) with certainty due to the ill-defined surface and weak interaction of the Au atoms. We do not have evidence for any transfer of Au particle from oxide (poorly interacted if prepared separately) to carbon but producing a rather homogenous material interfaces in close proximity (bottom).

a

b


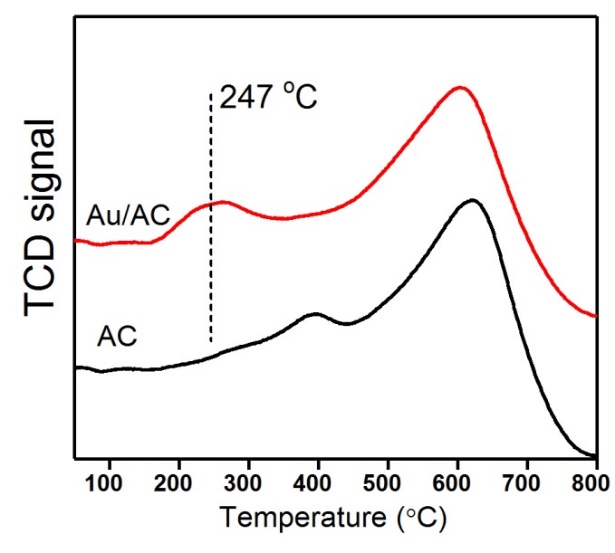


c

C_2_H_2_-TPD

Au/CeO_2_ⓜAC

CeO_2_

Au/AC

Au/CeO_2_

AC

Supplementary Figure 4. Temperature Programmed Reduction, TPR profiles of a Au/CeO_2_ and CeO_2_, b Au/AC and AC. The Au cations in as synthesized Au/CeO_2_ sample can be reduced to Au(0) under 158 ^o^C. However, the corresponding Au cations in Au/AC will be reduced to Au(0) at 247 ^o^C. The peaks from 400 to 600 ^o^C are attributed to the decarboxylations of the surface functional groups of AC. Thus, the Au reduction is taken place at lower temperature, when CeO_2_ is placed at intimate contact. This again indicates that the CeO_2_ can facilitate the redox of Au cations/Au(0).[^7^](#_ENREF_7) c C_2_H_2_ Temperature Programmed Desorption, TPD profiles of (left) CeO_2_, Au/CeO_2_, Au/CeO_2_/AC, AC, Au/AC (right) 13X, ZSM-5 and beta zeolites. As seen, CeO_2_ and Au/CeO_2_ did not take up much C_2_H_2_ but the use AC can give large uptake of C_2_H_2_ which was desorbed at about 150^o^C. Similarly, crystalline microporous supports such as 13X, ZSM-5 and beta zeolites also take up a significant C_2_H_2,_ which was desorbed at higher temperature (~200^o^C) showing the entrapment of C_2_H_2_ was stronger. Before the measurement of TPD, C_2_H_2_ adsorption was performed by passing a C_2_H_2_ stream (30 mL·min^-1^) over 0.2g of sample at 50 ºC for 30 min. TCD signal was monitored continuously when a flow of Ar (30 mL·min^-1^) was introduced to remove the gas-phase and weakly adsorbed C_2_H_2_ at a temperature ramp rate of 10 ºC·min^-1^ from 50 to 800 ºC.


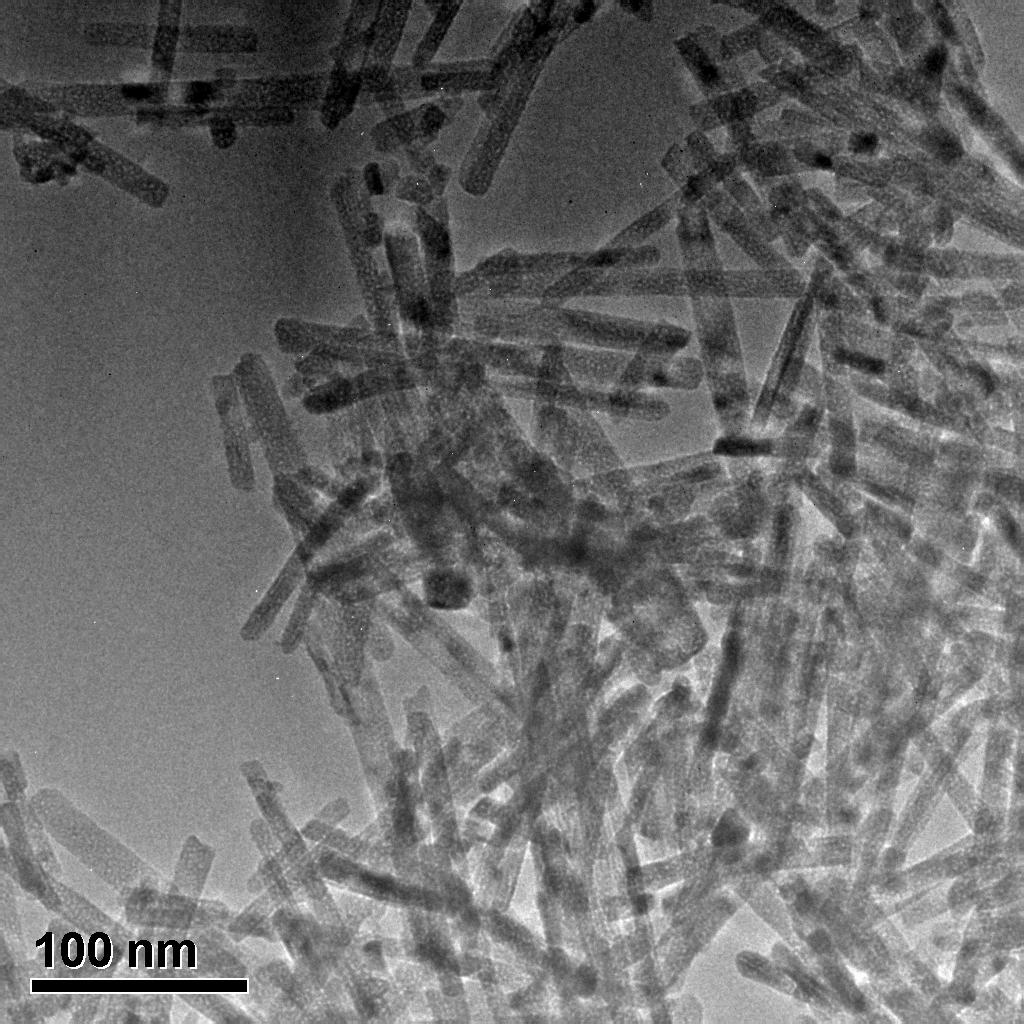
 Supplementary Figure 5. TEM of CeO_2_. CeO_2_ nanorod with (110) facet exposed.


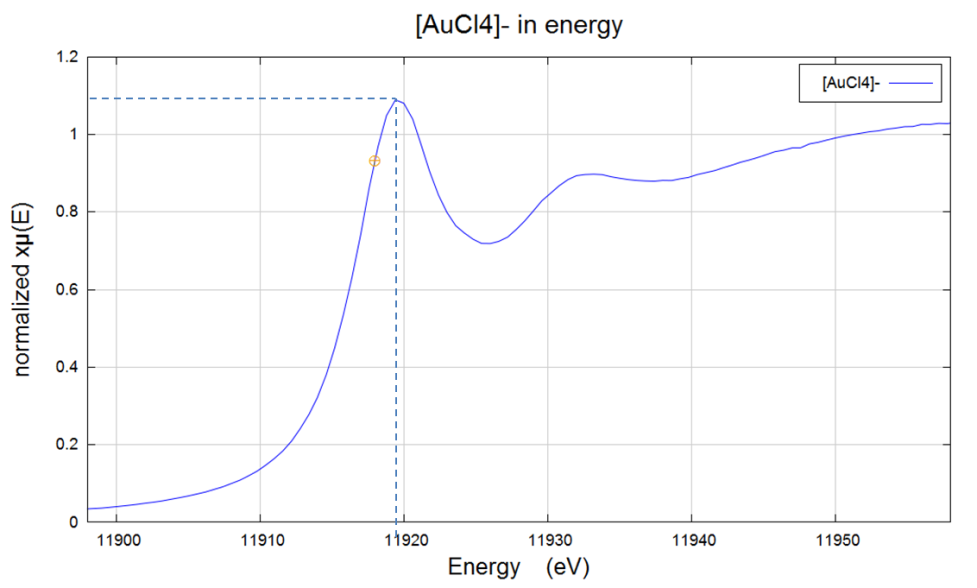


c

b

a


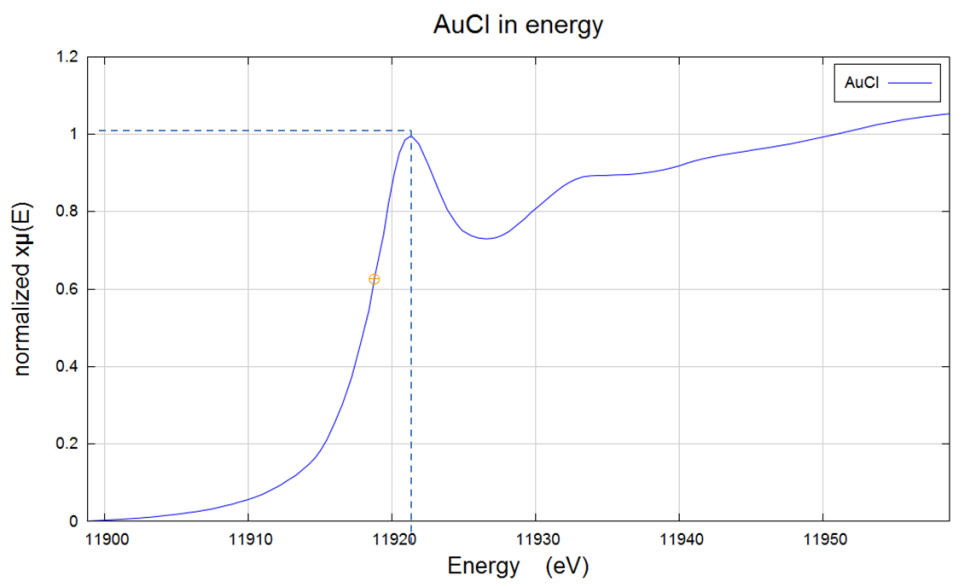


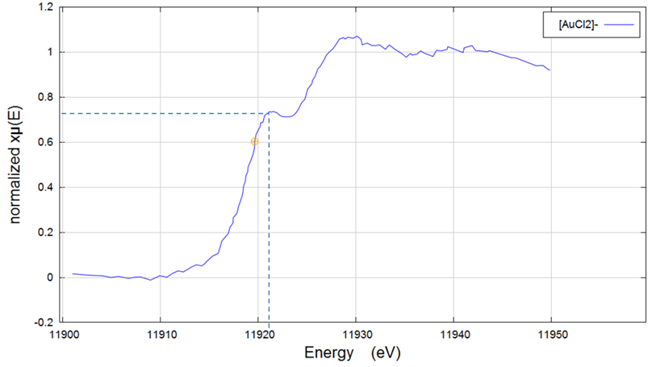


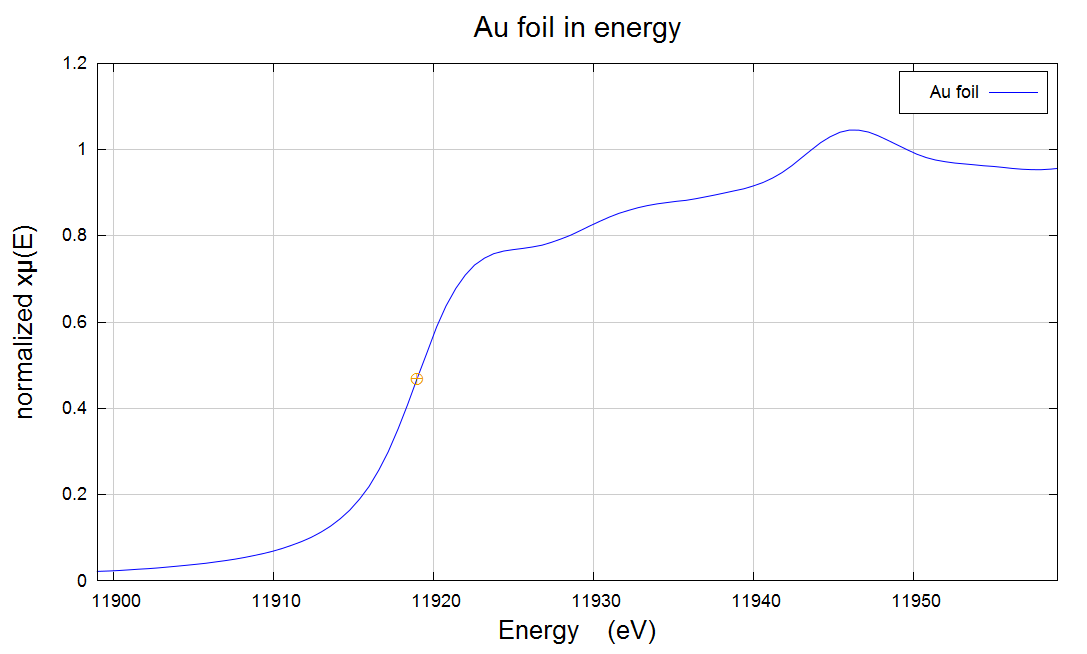


d

f
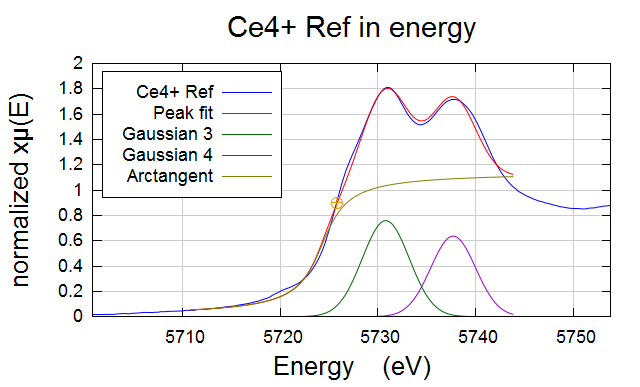


e


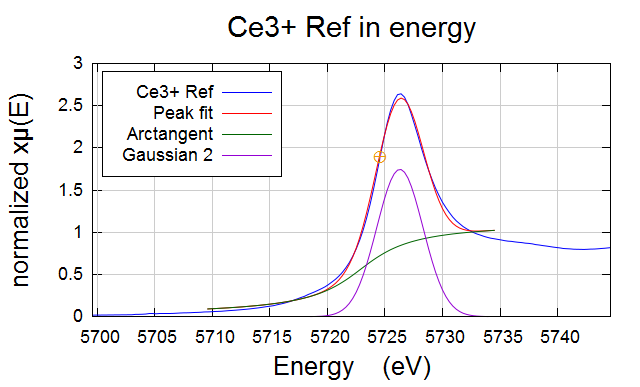


**Supplementary Figure 6.** Au L3-edge XANES region for the Au standards used: **a** [AuCl_4_]^-^, **b** Au(I) [AuCl_2_]^-^, **c** Au(I) [AuCl] and **d** Au(0) gold foil. Ce L3-edge XANES region for the Ce standards used: **e** Ce(IV) CeO_2_ and **f** Ce(III) CeCl_3_.

**a**


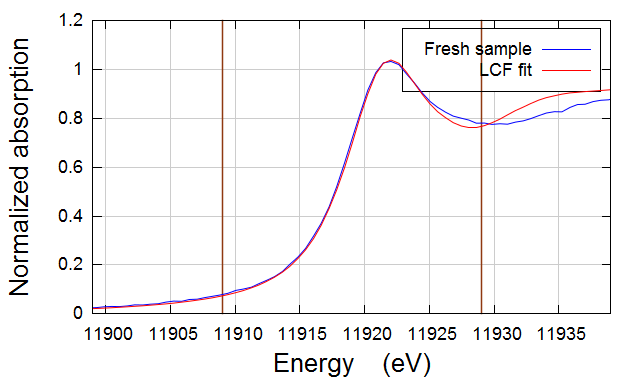


**b**


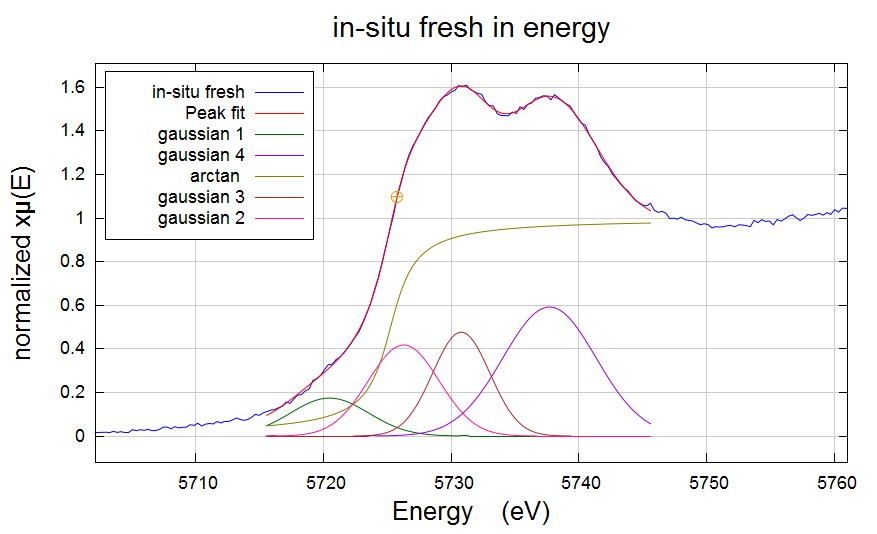


Supplementary Figure 7. XANES data fitting of the fresh Au/CeO_2_&AC at room temperature. a Au L3 edge (R-factor 0.003). b Ce L3 edge (R-factor 0.000052).

The linear combination fitting indicating around 0±5% contribution from Au(0), 74.9±4.7% from Au(III), 25.1±3.5% from Au(I). The multiple peak fitting of the white-line intensity of Ce edge showing that CeO_2_ under the conditions contains 73.6±1.2% of the signal of Ce(IV) and 26.4±1.1% Ce(III).


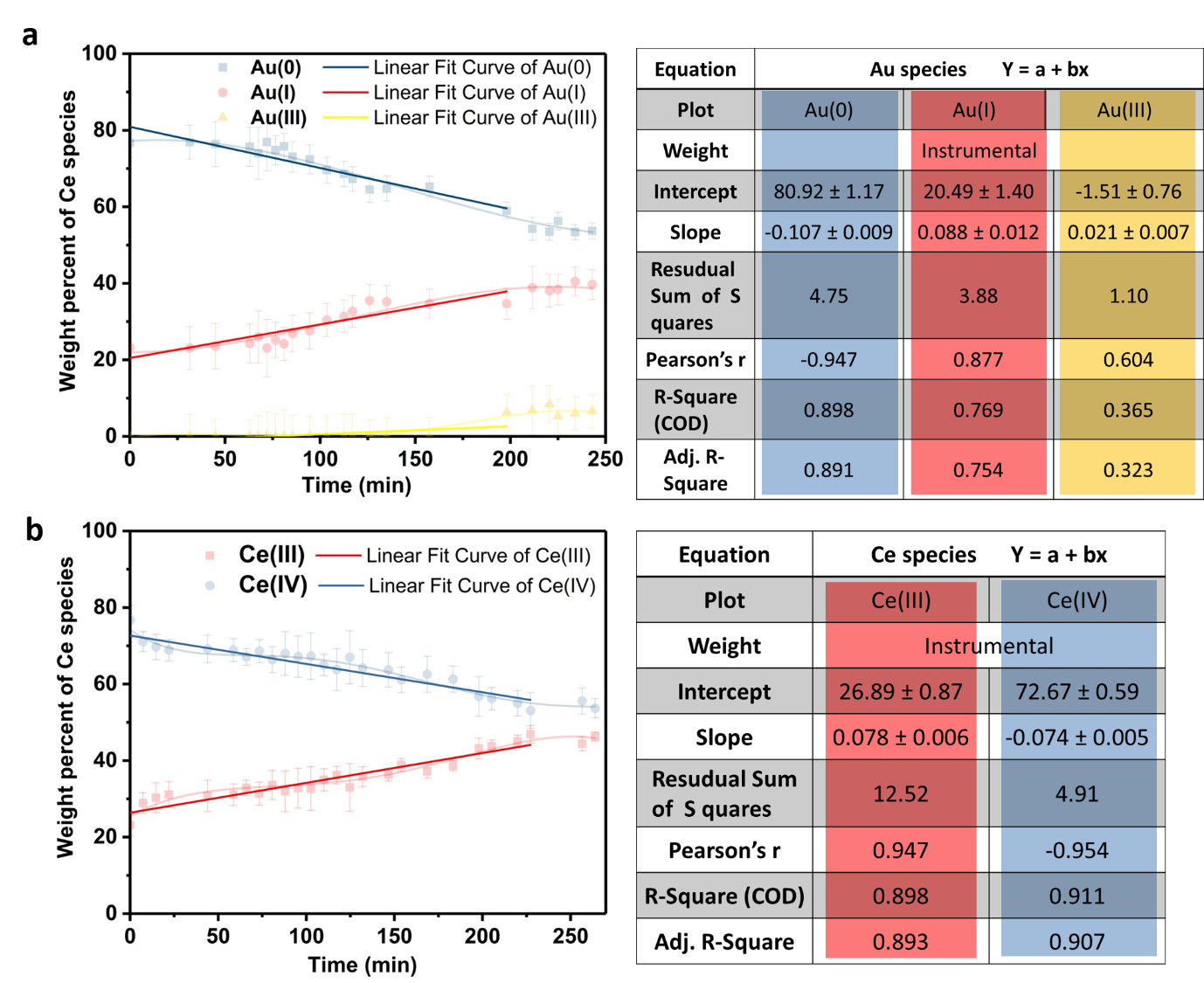


Supplementary Figure 8. The linear fitting of the XANES fitting values. a Au linear fitting and fitting information. b Ce linear fitting and fitting information.


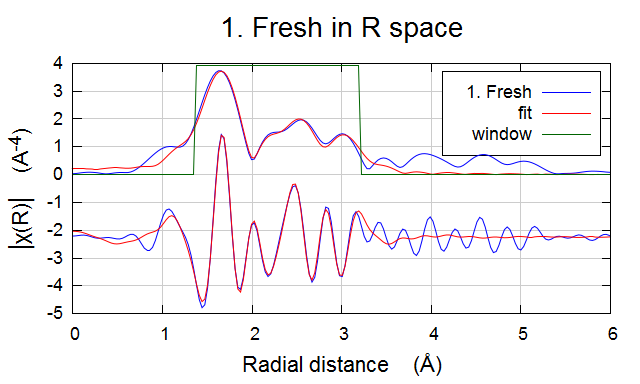


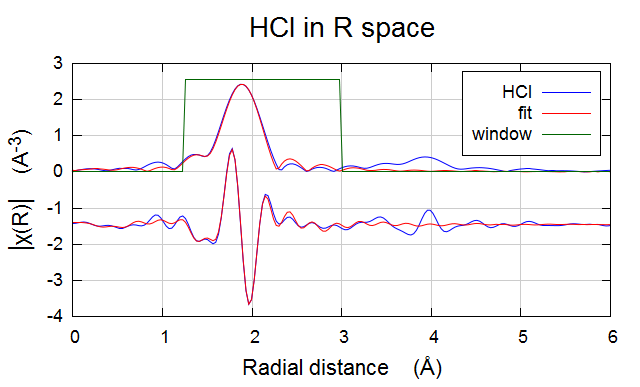


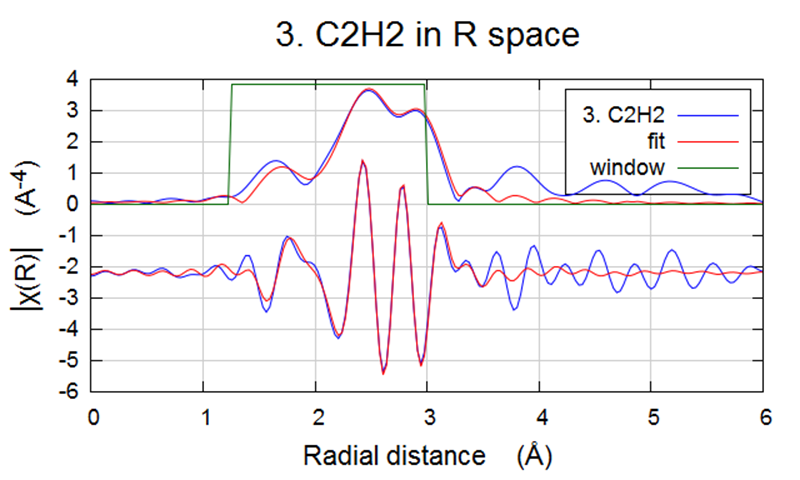

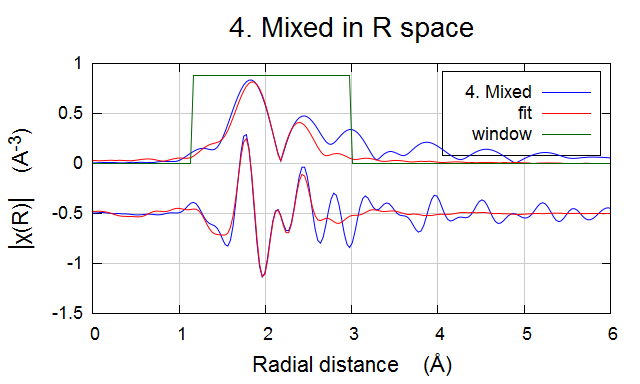


**Supplementary Figure 9.** *Ex-situ* EXAFS fitting picture.


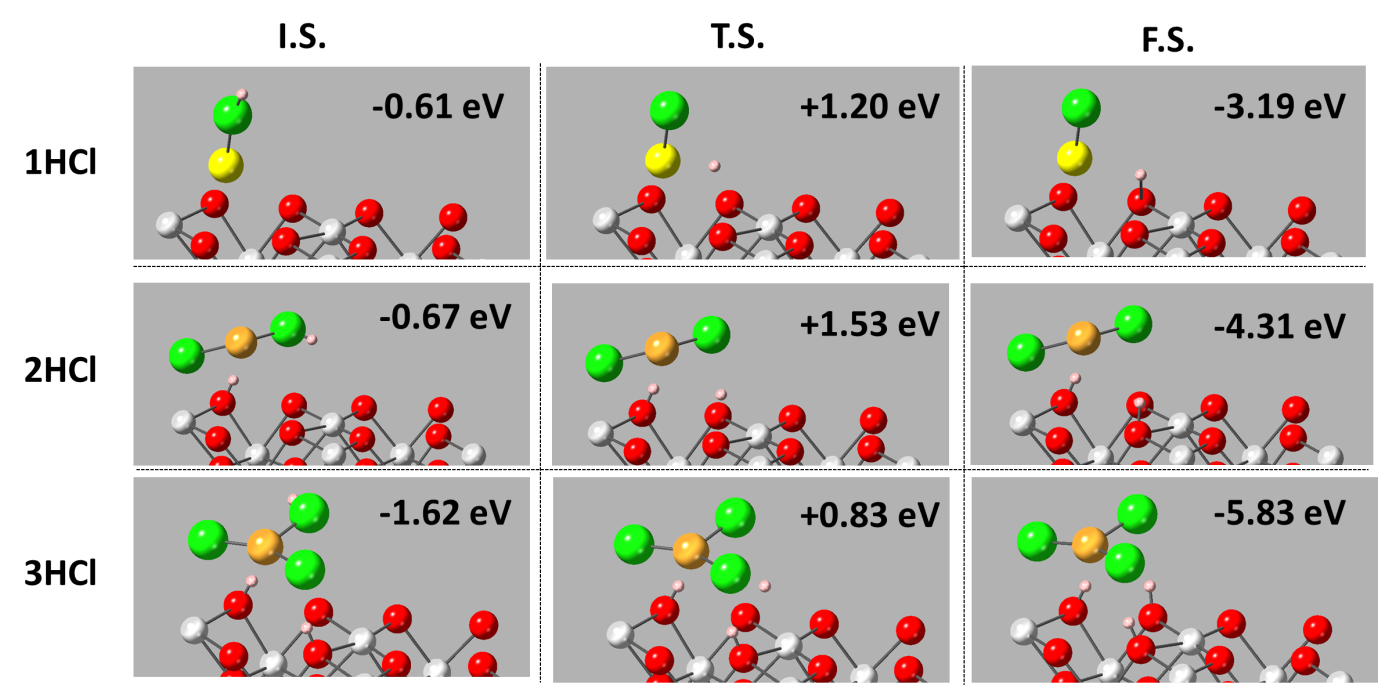


Supplementary Figure 10. The structures and relative energies of initial state (I.S.), transition state (T.S.) and final state (F.S.), while 1HCl, 2HCl and 3HCl dissociate over Au/CeO_2_.

Supplementary Tables

Supplementary Table 1. BET surface area of the catalysts

|  | BET Surface Area (m^2^·g^-1^) | Pore Volume (cm^3^·g^-1^) | Pore Diameter (nm) |
| --- | --- | --- | --- |
| CeO_2_-rods | 71.7 | 0.249 | 10.6 |
| Au/AC | 965 | 0.34 | 2.67 |
| Au/CeO_2_&AC | 1094.6 | 0.65 | 2.7 |
| Au/CeO_2_&13X | 732.1 | 0.43 | 2.30 |

Supplementary Table 2. Crystallographic data and details of C_2_H_2_ adsorbed 13X.

| Samples | | C_2_H_2_ adsorbed 13X | | | |  |
| --- | --- | --- | --- | --- | --- | --- |
| Crystal system | | Cubic | | | |  |
| Space group | | *Fd-3Z* | | | |  |
| Chemical formula | | Na_96_Si_96_Al_96_O_384_ | | | |  |
| 2θ range refinement (^o^) | | 2.5 – 50 | | | |  |
| Detector | | Multi-analyser crystals crystals | | | |  |
| Number of parameters | | 60 | | | |  |
| Number of hkls | | 765 | | | |  |
| Refinement methods | | Rietveld | | | |  |
| *a* (Å) | | 24.97072(3) | | | |  |
| *b* (Å) | | 24.97072(3) | | | |  |
| *c* (Å) | | 24.97072(3) | | | |  |
| *V* (Å^3^) | | 15570.165(62) | | | |  |
| *R_wp_ / R_p_ / R_exp_* (%) | | 9.981/7.315/2.429 | | | |  |
| Wavelength (Å) | | 0.825775(2) | | | |  |
| 2θ Zero point (°) | | -0.000753(2) | | | |  |
| Gof χ^2^ | | 4.10 | | | |  |
|  |  |  |  |  | |  |

Supplementary Table 3. Atomic parameters from the Rietveld refinement of C_2_H_2_ adsorbed 13X at room temperature.

| Species | Atom | *X* | *Y* | *Z* | SOF | B_eq_ (Å^2^) | multiplicity |
| --- | --- | --- | --- | --- | --- | --- | --- |
| Zeolite framework | Si1 | -0.0537 | 0.0346 | 0.1244 | 1 | 0.299(29) | 96 |
|  | Al1 | -0.0539 | 0.1225 | 0.0362 | 1 | 1.884(22) | 96 |
|  | O1 | -0.1063 | -.0010 | 0.1111 | 1 | 2.827(33) | 96 |
|  | O2 | -0.0051 | -.0031 | 0.1452 | 1 | 2.827(33) | 96 |
|  | O3 | -0.0352 | 0.0646 | 0.0686 | 1 | 2.827(33) | 96 |
|  | O4 | -0.0677 | 0.0762 | 0.1711 | 1 | 2.827(33) | 96 |
| Na | Na1 | 0.053 | 0.053 | 0.053 | 0.689(3) | 2.7(1) | 32 |
|  | Na2 | 0.2283 | 0.2283 | 0.2283 | 0.994(3) | 3.1(1) | 32 |
|  | Na3 | 0.875 | 0.875 | 0.875 | 0.513(7) | 19.6(8) | 8 |
| Acetylene 1 |  |  |  |  |  |  |  |
|  | C1 | 4.3203 | 4.6841 | 6.0728 | 1.78(1) | 1.9(2) | 16 |
|  | C2 | 4.3298 | 4.6563 | 6.1108 | 1.78(1) | 1.9(2) | 16 |
| Acetylene 2 |  |  |  |  |  |  |  |
|  | C1 | 3.7143 | 4.8241 | 6.5527 | 1.88(1) | 4.9(3) | 16 |
|  | C2 | 3.7079 | 4.8165 | 6.5057 | 1.88(1) | 4.9(3) | 16 |

**Supplementary Table 4. *In situ* XANES results and fitting parameters of Au/CeO_2_&AC under reaction condition.**

|  |  | Quantification (%) | | | | R factor | ΔE^0^ | | |
| --- | --- | --- | --- | --- | --- | --- | --- | --- | --- |
|  | Time (min) | Au(0) | Au(I) | | Au(III) |  | Au(0) | Au(I) | Au(III) |
| Au | 0 | 76.7(3.8) | 23.3(5.2) | | 0.0(5.5) | 0.00132 | -0.479 | | |
|  | 31.5 | 76.9(4.4) | 23.1(5.6) | | 0.0(5.8) | 0.00213 | -0.344 | | |
|  | 45 | 76.4(5.9) | 23.6(5.9) | | 0.0(4.5) | 0.00392 | -0.136 | | |
|  | 63 | 75.7(5.1) | 24.3(5.1) | | 0.0(4.4) | 0.00281 | -0.641 | | |
|  | 67.5 | 74.0(6.8) | 26.0(6.8) | | 0.0(5.4) | 0.00538 | -0.513 | | |
|  | 72 | 76.9(7.5) | 23.1(7.5) | | 0.0(6.4) | 0.00538 | -0.520 | | |
|  | 76.5 | 74.8(3.8) | 25.2(4.6) | | 0.0(6.0) | 0.00654 | -0.264 | | |
|  | 81 | 75.8(3.4) | 24.2(4.3) | | 0.0(6.9) | 0.004 | -0.395 | | |
|  | 85.5 | 73.1(4.0) | 26.9(4.8) | | 0.0(5.5) | 0.00315 | -0.276 | | |
|  | 94.5 | 72.4(3.8) | 27.6(4.7) | | 0.0(5.3) | 0.004 | -0.252 | | |
|  | 103.5 | 69.6(3.4) | 30.4(4.3) | | 0.0(5.4) | 0.00323 | -0.279 | | |
|  | 112.5 | 68.6(3.3) | 31.4(4.2) | | 0.0(5.8) | 0.00302 | -0.494 | | |
|  | 117 | 67.3(3.2) | 32.7(4.1) | | 0.0(5.9) | 0.0028 | -0.370 | | |
|  | 126 | 64.5(3.3) | 35.5(4.2) | | 0.0(4.3) | 0.00318 | -0.567 | | |
|  | 135 | 64.8(3.3) | 35.2(4.2) | | 0.0(4.8) | 0.00305 | -0.454 | | |
|  | 157.5 | 65.3(2.7) | 34.7(3.8) | | 0.0(4.9) | 0.00208 | -0.683 | | |
|  | 198 | 59.0(2.2) | 34.7(4.1) | | 6.3(4.6) | 0.00152 | -0.656 | | |
|  | 211.5 | 54.3(3.0) | 38.9(5.5) | | 6.8(6.3) | 0.00262 | -0.424 | | |
|  | 220.5 | 53.5(2.2) | 38.1(4.3) | | 8.4(4.8) | 0.00145 | -0.544 | | |
|  | 225 | 56.3(2.3) | 38.4(4.0) | | 5.2(4.6) | 0.00155 | -0.810 | | |
|  | 234 | 53.4(2.0) | 40.5(3.8) | | 6.0(4.3) | 0.00128 | -0.742 | | |
|  | 243 | 53.7(2.1) | 39.7(3.9) | | 6.6(4.4) | 0.00129 | -0.640 | | |
|  | | | | | | | | | |
|  |  | Quantification (%) | | | | R factor | | | |
|  | Time (min) | Ce(III) | | Ce(IV) | |  |  |  |  |
| Ce | 0 | 23.2(2.9) | | 76.8(3.8) | | 5.945E-4 | | | |
|  | 7.33 | 28.9(2.7) | | 71.1(2.6) | | 1.813E-4 | | | |
|  | 14.66 | 30.3(3.9) | | 69.7(3.3) | | 2.642E-4 | | | |
|  | 21.99 | 31.1(3.4) | | 68.9(2.8) | | 1.736E-4 | | | |
|  | 43.98 | 30.8(4.1) | | 69.2(3.6) | | 1.738E-4 | | | |
|  | 58.64 | 31.1(2.8) | | 68.9(3.0) | | 1.981E-4 | | | |
|  | 65.97 | 32.9(2.0) | | 67.1(2.2) | | 2.035E-4 | | | |
|  | 73.30 | 31.4(3.0) | | 68.6(3.0) | | 1.804E-4 | | | |
|  | 80.63 | 33.6(3.8) | | 66.4(3.4) | | 1.916E-4 | | | |
|  | 87.96 | 32.0(5.3) | | 68.0(5.7) | | 1.248E-4 | | | |
|  | 95.29 | 32.8(5.2) | | 67.2(5.4) | | 1.235E-4 | | | |
|  | 102.62 | 32.7(5.7) | | 67.3(6.1) | | 1.434E-4 | | | |
|  | 109.95 | 35.0(2.9) | | 65.0(2.8) | | 2.120E-4 | | | |
|  | 117.28 | 36.2(2.3) | | 63.8(5.5) | | 1.461E-4 | | | |
|  | 124.61 | 33.0(6.2) | | 67.0(6.9) | | 1.809E-4 | | | |
|  | 131.94 | 35.8(2.6) | | 64.2(4.8) | | 1.671E-4 | | | |
|  | 146.6 | 36.3(1.6) | | 63.7(4.5) | | 2.312E-4 | | | |
|  | 153.93 | 38.9(1.7) | | 61.1(3.3) | | 1.614E-4 | | | |
|  | 168.59 | 37.2(1.8) | | 62.8(4.7) | | 1.773E-4 | | | |
|  | 183.25 | 38.7(1.3) | | 61.3(3.4) | | 1.450E-4 | | | |
|  | 197.91 | 43.2(2.7) | | 56.8(5.2) | | 1.952E-4 | | | |
|  | 205.24 | 43.8(1.6) | | 56.2(2.9) | | 1.968E-4 | | | |
|  | 219.9 | 45.0(1.7) | | 55.0(3.3) | | 1.918E-4 | | | |
|  | 227.23 | 46.9(2.3) | | 53.1(4.7) | | 1.800E-4 | | | |
|  | 256.55 | 44.4(1.8) | | 55.6(3.4) | | 2.224E-4 | | | |
|  | 263.88 | 46.3(1.2) | | 53.7(2.5) | | 2.199E-4 | | | |

Supplementary Table 5. *Ex-situ* EXAFS results and fitting parameters

| **Ex-situ** | **EXAFS** | | | | |
| --- | --- | --- | --- | --- | --- |
|  | **Scattering Path** | **Bond Length (Å)** | **CN** | **Debye-Waller factor** | **Enot* (eV)** |
| **Fresh Sample**  **(N_2_, RT)** | **Au-Cl**  **Au-Au** | **2.13 ± 0.01**  **2.90 ± 0.01** | **2.0 ± 0.1**  **7.7 ± 0.4** | **0.009 ± 0.002**  **0.015 ± 0.002** | **1.6**  **7.8** |
| **HCl**  **Passing** | **Au-Cl** | **2.27 ± 0.01** | **3.5 ± 0.2** | **0.002 ± 0.001** | **5.1** |
| **C_2_H_2_**  **Passing** | **Au-Cl**  **Au-C**  **Au-Au** | **2.14 ± 0.01**  **2.38 ± 0.02**  **2.83 ± 0.01** | **0.4 ± 0.1**  **1.4 ± 0.3**  **9.0 ± 0.4** | **0.003 ± 0.001**  **0.009 ± 0.002**  **0.011 ± 0.001** | **3.5** |
| **HCl/C_2_H_2_ passing** | **Au-Cl**  **Au-O** | **2.26 ± 0.02**  **2.87 ± 0.02** | **1.4 ± 0.3**  **1.5 ± 0.6** | **0.003 ± 0.001**  **0.001 ± 0.001** | **7.05** |

Notably, EXAFS derived Au coordination number with prolonged data acquisition under ex-situ conditions are also consistent with the trend of XANES. As Table S5 shown, Au-Cl and Au-Au distances are resolved in the fresh sample and the correlated coordination numbers (CN) are determined to be 2.0 and 7.7, respectively. It indicates that the mixture contains Au coordinated with three Cl (as Au(III)-like species) and one with Cl (as Au(I)-like species) on surface and a small quantity of Au(0) as Au cluster/nanoparticle by comparing the data with the Au foil CN of 12 as also shown by the XANES. Interestingly, by placing this sample in pure HCl for extended time, no Au-Au distance is detected anymore and the Au-Cl CN increases to 3.5, presumably the Au cluster/nanoparticle is rapidly dispersed and oxidised when in contact with CeO_2_ (110) and HCl as [AuCl_4_]^-^ through exhaustive Cl replacement of O (totally oxidised by pure HCl). However, the Au-Au distance resumes to 9.0 (larger Au nanoparticle) and Au-Cl CN decreases to 0.4 (Au(I)-like species) after passing the pure C_2_H_2_ (reductant) with a little Au-C distance presented. When exposing the sample in contact with the mixture gas with HCl : C_2_H_2_ = 2 : 1, the formation of Au(I)-Cl like species (CN is found to be 1.4) from [Au(0)-O_4_] due to mediating role of Ce(III) as described in Fig. 4C in the main text with Au-O at 1.5 at steady state. This observation indicates the oxidation states of Au can be changed among Au(0), Au(I) and Au(III) over the CeO_2_ (110) surface dependent on the composition of substrate gases. This finding reinforces the Au species is re-generable as the active and dispersive Au(0)/Au(I) mediated by the CeO_2_ under the substrate gas mixture, even it had been extensively reduced to Au(0) aggregate/nanoparticle.

**Supplementary References**

1 Mai, H.-X. *et al.* Shape-selective synthesis and oxygen storage behavior of ceria nanopolyhedra, nanorods, and nanocubes. *J. Phys. Chem. B* **109**, 24380-24385 (2005).

2 Chen, B.-B., Shi, C., Crocker, M., Wang, Y. & Zhu, A.-M. Catalytic removal of formaldehyde at room temperature over supported gold catalysts. *Appl. Catal., B* **132**, 245-255 (2013).

3 Malta, G. *et al.* Identification of single-site gold catalysis in acetylene hydrochlorination. *Science* **355**, 1399-1402 (2017).

4 Yang, Z. *et al.* Sulfur-doped graphene as an efficient metal-free cathode catalyst for oxygen reduction. *ACS Nano* **6**, 205-211 (2012).

5 Ke, J. H. *et al.* Yttrium chloride-modified Au/AC catalysts for acetylene hydrochlorination with improved activity and stability. *Journal Of Rare Earths* **35**, 1083-1091 (2017).

6 Hoshiya, N. *et al.* Sulfur modification of Au via treatment with piranha solution provides low-pd releasing and recyclable pd material, sapd. *J. Amer. Chem. Soc.* **132**, 7270-7272 (2010).

7 Conte, M. *et al.* Aqua regia activated Au/C catalysts for the hydrochlorination of acetylene. *J. Catal.* **297**, 128-136 (2013).

8 Thompson, S. P. *et al.* Beamline I11 at Diamond: A new instrument for high resolution powder diffraction. *Rev. Sci. Instrum.* **80** (2009).

9 Butikova, I. K., Shepelev, Y. F. & Smolin, Y. I. Crystal-structure of csnax-zeolite in hydrated and dehydrated forms. *Kristallografiya* **34**, 1141-1145 (1989).

10 Bianconi, A. *et al.* Specific intermediate-valence state of insulating 4f compounds detected by X-ray absorption. *Phys. Rev. B: Condens. Matter* **35**, 806-812 (1987).

11 Dexpert, H. *et al.* X-ray absorption studies of CeO_2_, PrO_2_, and TbO_2_ .2. rare-earth valence state by liii absorption edges. *Phys. Rev. B: Condens. Matter* **36**, 1750-1753 (1987).

12 Karnatak, R. C., Esteva, J. M. & Dexpert, H. x-ray absorption studies of CeO_2_, PrO_2_, and TbO_2_ .1. Manifestation of localized and extended f-states in the 3d absorption-spectra. *Phys. Rev. B: Condens. Matter* **36**, 1745-1749 (1987).

13 Blochl, P. E. Projector augmented-wave method. *Phys. Rev. B: Condens. Matter* **50**, 17953-17979 (1994).

14 Kresse, G. & Joubert, D. From ultrasoft pseudopotentials to the projector augmented-wave method. *Phys. Rev. B: Condens. Matter* **59**, 1758-1775 (1999).

15 Payne, M. C., Teter, M. P., Allan, D. C., Arias, T. A. & Joannopoulos, J. D. Iterative minimization techniques for abinitio total-energy calculations - molecular-dynamics and conjugate gradients. *‎Rev. Mod. Phys.* **64**, 1045-1097 (1992).

16 Vanderbilt, D. Soft self-consistent pseudopotentials in a generalized eigenvalue formalism. *Phys. Rev. B: Condens. Matter* **41**, 7892-7895 (1990).

17 Perdew, J. P. *et al.* Atoms, molecules, solids, and surfaces - applications of the generalized gradient approximation for exchange and correlation. *Phys. Rev. B: Condens. Matter* **46**, 6671-6687 (1992).

18 Perdew, J. P. Electronic Structure of Solids 1991. *Aka- demie Verlag, Berlin, 1991*.

19 Kresse, G. & Furthmuller, J. Efficient iterative schemes for ab initio total-energy calculations using a plane-wave basis set. *Phys. Rev. B: Condens. Matter* **54**, 11169-11186 (1996).

20 Kresse, G. & Hafner, J. Abinitio molecular-dynamics for liquid-metals. *Phys. Rev. B: Condens. Matter* **47**, 558-561 (1993).

21 Henkelman, G., Uberuaga, B. P. & Jonsson, H. A climbing image nudged elastic band method for finding saddle points and minimum energy paths. *J. Chem. Phys.* **113**, 9901-9904 (2000).

22 Mills, G., Jonsson, H. & Schenter, G. K. Reversible work transition-state theory - application to dissociative adsorption of hydrogen. *‎Surf. Sci.* **324**, 305-337 (1995).

23 Ulitsky, A. & Elber, R. A new technique to calculate steepest descent paths in flexible polyatomic systems. *J. Chem. Phys.* **92**, 1510-1511 (1990).
