# Supplementary material for: Self- regeneration of Au/CeO2 based catalysts with enhanced activity and ultra-stability for acetylene hydrochlorination
Source: Nat Commun. 2019 Feb 22;10:914. doi: 10.1038/s41467-019-08827-5 (PMC6385229; doi:10.1038/s41467-019-08827-5)
Supplement: Supplementary file 3 — Description of Additional Supplementary Files [file 41467_2019_8827_MOESM3_ESM.docx]

**Description of Additional Supplementary Files**

File Name: Supplementary Movie 1

Description: Au atoms on ceria
