# Supplementary figures and images for: Self- regeneration of Au/CeO2 based catalysts with enhanced activity and ultra-stability for acetylene hydrochlorination
Source: Nat Commun. 2019 Feb 22;10:914. doi: 10.1038/s41467-019-08827-5 (PMC6385229; doi:10.1038/s41467-019-08827-5)

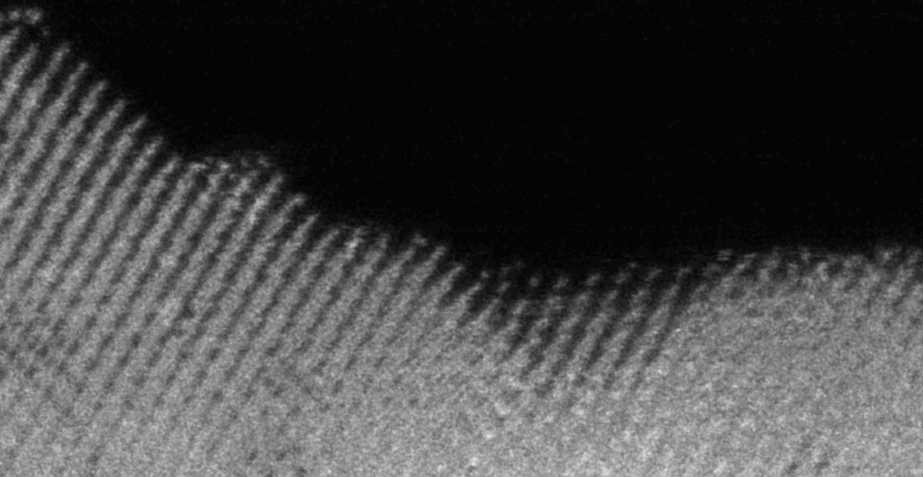

Supplement: Supplementary file 4 — Supplementary Movie 1 [file 41467_2019_8827_MOESM4_ESM.gif]
