# Supplementary material for: Self- regeneration of Au/CeO2 based catalysts with enhanced activity and ultra-stability for acetylene hydrochlorination
Source: Nat Commun. 2019 Feb 22;10:914. doi: 10.1038/s41467-019-08827-5 (PMC6385229; doi:10.1038/s41467-019-08827-5)
Supplement: Supplementary file 2 — Peer Review File [file 41467_2019_8827_MOESM2_ESM.pdf]

REVIEWERS' COMMENTS:

Reviewer #1 (Remarks to the Author):

This manuscript has been reviewed when it was firstly submitted. In this revised manuscript, the authors have made substantial improvement and most of the issues raised by myself (and the other reviewers) have been properly solved. In general, I support its acceptance to be published in Nature Communications.

Reviewer #2 (Remarks to the Author):

As referee 1 of the original submission I feel the author has dismissed my comments rather than address them. While the thiosulfate route is now mentioned the authors still imply this deactivates. The published literature shows this is not the case. Hence this paper is solving a non existent problem at the commercial level.
This needs to be fully recognised. The addition of Ce is indeed intersting but it adds to the complexity. In reality this permits another way to make a stable Au catalyst for VCM production, it is more complicated but it works. That should be the message.

Reviewer #3 (Remarks to the Author):

The authors have improved the manuscript greatly according to the reviewers' comments. They intensively studied the effects of ceria on the activity of gold catalysts, and concluded that Au(0) would be directly involved in catalysis for acetylene hydrochlorination instead of aggregations, illustrating that the strong mediating properties of Ce(IV)/Ce(III) could enhance the activity and stability of Au catalysts. The results are interesting, and would push forward the progress of exploring highly efficient non-mercuric catalysts to produce vinyl chloride. It is deserved to be published in the journal of Nature Communication.

REVIEWERS' COMMENTS:

Reviewer #1 (Remarks to the Author):

This manuscript has been reviewed when it was firstly submitted. In this revised manuscript, the authors have made substantial improvement and most of the issues raised by myself (and the other reviewers) have been properly solved. In general, I support its acceptance to be published in Nature Communications.

Response to reviewer 1:
We thank the reviewer 1 who accepted our improvement and modification of this manuscript.

Reviewer #2 (Remarks to the Author):

As referee 1 of the original submission I feel the author has dismissed my comments rather than address them. While the thiosulfate route is now mentioned the authors still imply this deactivates. The published literature shows this is not the case. Hence this paper is solving a non existent problem at the commercial level.
This needs to be fully recognised. The addition of Ce is indeed intersting but it adds to the complexity. In reality this permits another way to make a stable Au catalyst for VCM production, it is more complicated but it works. That should be the message.

Response to reviewer 2:
We thank the reviewer 2 (original referee 1) who agreed with us that the Ce can offer a new way to stabilize Au for VCM production. We are pleased that he/she recommended the publication of our paper.

In light of the comment of this reviewer, we have modified our introduction to acknowledge the commercial success by the use of thiosulfate as follows: "The adaptation of chelating soft donor atoms (e.g. thiosulfate, thiocyanate, thiourea, cyanides) and later nitrogen-containing ligands (i.e. phenanthroline ligands) have been shown to be effective to reduce over-reduction of cationic Au atoms and improve the activity and stability of Au(I)/Au(III) catalyst mixture at the acceptable industrial scale[29-30]. Despite the commercial success by this approach there is still an ongoing research in searching alternative stabilization means to reduce the progressive deactivation.

Reviewer #3 (Remarks to the Author):

The authors have improved the manuscript greatly according to the reviewers' comments. They intensively studied the effects of ceria on the activity of gold catalysts, and concluded that Au(0) would be directly involved in catalysis for acetylene hydrochlorination instead of aggregations, illustrating that the strong mediating properties of Ce(IV)/Ce(III) could enhance the activity and stability of Au catalysts. The results are interesting, and would push forward the progress of exploring highly efficient non-mercuric catalysts to produce vinyl chloride. It is deserved to be

published in the journal of Nature Communication.

Response to reviewer 3:

We thank the reviewer 3 who found our work interesting. He/she agreed with us that this work could push forward the progress of exploring highly efficient non-mercuric catalysts to produce vinyl chloride.
